# The upregulation of VGF enhances the progression of oral squamous carcinoma

**DOI:** 10.1186/s12935-024-03301-9

**Published:** 2024-03-25

**Authors:** Chung-Hsien Chou, Chun-Han Yen, Chung-Ji Liu, Hsi-Feng Tu, Shu-Chun Lin, Kuo-Wei Chang

**Affiliations:** 1 Institute of Oral Biology, College of Dentistry, National Yang Ming Chiao Tung University, Taipei, Taiwan; 2 Department of Dentistry, College of Dentistry, National Yang Ming Chiao Tung University, Taipei, Taiwan; 3 Department of Stomatology, Taipei Mackay Memorial Hospital, Taipei, Taiwan; 4 Department of Stomatology, Taipei Veterans General Hospital, Taipei, Taiwan

**Keywords:** IL23R, miR-432-5p, Oral cancer, Tumor microenvironment, VGF

## Abstract

**Background:**

Oral squamous cell carcinoma (OSCC) is a prevalent neoplasm worldwide, necessitating a deeper understanding of its pathogenesis. VGF nerve growth factor inducible (VGF), a neuropeptide, plays critical roles in nerve and endocrine cell regulation.

**Methods:**

In this study, the TCGA datasets were initially screened, identifying the upregulation of VGF in various malignancies. We focused on OSCC cell lines, identifying the suppressor mRNA miR-432-5p as a negative regulator of VGF. Additionally, we examined the prognostic value of VGF expression in OSCC tumors and its impact on cellular functions.

**Results:**

VGF expression was found to be an independent prognostic predictor in OSCC tumors. Cells expressing VGF exhibited increased oncogenicity, influencing the proliferation and migration of oral mucosal fibroblast. Transcriptome analysis revealed associations between VGF and various pathological processes, including malignancies, exosome release, fibrosis, cell cycle disruption, and tumor immune suppression. Moreover, IL23R expression, a favorable OSCC prognostic factor, was inversely correlated with VGF expression. Exogenous IL23R expression was found to suppress VGF-associated mobility phenotypes.

**Conclusions:**

This study highlights the multifaceted role of VGF in OSCC pathogenesis and introduces the miR-432-5p-VGF-IL23R regulatory axis as a critical mediator. The combined expression of VGF and IL23R emerges as a potent predictor of survival in oral carcinoma cases, suggesting potential implications for future therapeutic strategies.

**Supplementary Information:**

The online version contains supplementary material available at 10.1186/s12935-024-03301-9.

## Background

Head and neck squamous cell carcinoma (HNSCC), including oral SCC (OSCC), are among the highly prevalent malignancies worldwide [1–5]. Our research has focused on elucidating the molecular mechanisms involved in the OSCC pathogenesis. miRNAs bind to sequences in the 3’UTR of targeted transcripts, thereby mediating RNA interference [2, 4–6]. Our previous works have elucidated the aberrances of miRNAs and their targets during oral neoplastic process [5]. Besides, we have also demonstrated the activities of NUMB gene in abrogating oncogenicity and aerobic respiration in OSCC [2, 3]. Several oncogenic miRNAs, including miR-31/82/146a/192, co-target NUMB to drive oncogenicity in HNSCC or OSCC [2, 3, 6].

Nerve growth factor inducible (VGF, NGF inducible, non-acronym) was initially identified as a stress-responsive ∼68-kDa nerve or neuroendocrine-specific prohormone [7–10]. VGF precursor polypeptide and various of its derived secretory peptides drive pluripotent activities in the differentiation, physiological signaling, or disease processes of neuronal, neuroendocrine, and endocrine cells; energy balance, organ injury, and the homeostasis of psychosomatic state [10–19]. Peptides derived from precursor VGF demonstrate tissue specificity and mediate differential responses [19]. VGF also maintains the stemness and self-renewal of glioma cells through the autocrine or paracrine modulation [20]. Apart from the tissue-specific secretion, exogenous VGF expression resulted in the formation of a cytosolic grain-like structure and induced VGF secretion in NIH 3T3 mesenchymal cells [21]. Secretion of VGF was also identified in thyroid follicular epithelial cells [22]. As the secreted VGF has been detected in neuroendocrine carcinoma-derived lung cells [23], it was further proven to be a critical factor in mediating drug resistance, epithelial-to-mesenchymal transition, and patient survival in lung malignancies exhibiting neuroendocrine features [24, 25]. Silencing of VGF due to the methylation of promoter has been identified in urothelial cell carcinoma and ovarian carcinoma [26]. However, VGF was defined as a driver of radiation resistance and an unfavorable prognostic predictor in prostate cancer patients receiving radiotherapy [27, 28]. Since the functions of VGF in the neoplastic process are largely unknown and our previous works delineated the robust opposite expression between VGF expression and NUMB expression in OSCC [2, 3, 6], this study aims to explore the pathogenetic activities of VGF in HNSCC/OSCC.

VGF regulates the proliferation of lymphocytes [29]. After that, lines of evidence have demonstrated VGF as one of the differentially expressed genes for annotating immune cell infiltration profile and prognostic values in malignancies such as non-small cell lung carcinoma (NSCLC), colorectal carcinoma (CRC) and thyroid carcinoma [30–32]. It is important to delineate the potential impacts of VGF on the tumor immune microenvironment (TIME) and patient survival. A recent study has shown that the protein translation of the VGF transcript is repressed by its 3′UTR [17]. However, the epigenetic regulation of miRNAs by targeting the 3’UTR of VGF remains elusive. Interleukin (IL) 23 (IL23) belongs to the IL12 cytokine family, which plays essential roles in modulating TIME and inflammatory disorders [33]. IL23, produced by activated dendritic cells and macrophages, targets immune and epithelial cells to activate IL23R signaling, thereby mediating colorectal inflammation and CRC tumorigenesis [34, 35]. However, the activity of IR23R in neoplastic pathogenesis, including HNSCC and OSCC, remains controversial [36–38]. This work identifies the crucial oncogenic roles of VGF in the oral carcinogenesis process and ascertains the co-play of miR-432-5p and IL23R with VGF in OSCC.

## Methods

### Cell culture, reagents, and phenotypic assays

The SAS, FaDu, OECM1 OSCC cell lines, and H1299 NSCLC cell lines were cultured as previously described [2, 24]. The HEK293T cell line was utilized for virus production for infection. Oral mucosal fibroblast-1 (OMF-1) was a kind gift from Prof. Li, W.C. All cell lines have been authenticated using Short Tandem Repeats (STR) profiling within three years. All cultivated conditions were mycoplasma-free. mirVana miR-432-5p mimic (Cat No. #4,464,066) and scr control were purchased from ThermoFisher Scientific (Waltham, MA, USA). Protein secretion was inhibited by BFA (Befeldin A; Sigma-Aldrich, St Louise, MO, USA) [39]. The Transfectin™ Reagent (BioRad, Hercules, CA, USA) was employed for transfection. The cell viability was assayed with 3-(4,5-dimethylthiazol-2-yl)-2,5-diphenyltetrazolium bromide or trypan blue (both reagents purchased from Sigma-Aldrich). The fold changes in viability across different time points were plotted and transformed into semi-log graphs. The differences in growth curves and population doubling time (PDT) in the exponential growth phase were analyzed. Cell migration, invasion, and co-culture were analyzed using transwell (Millicell Hanging Cell Culture Insert; Merck, Darmstadt, Germany) assays [2]. Unless specified, all other materials were purchased from Sigma-Aldrich.

### Samples and RNA-Seq

Samples from 54 OSCC patients and 28 noncancerous matched normal tissue were collected at Taipei Mackay Memorial Hospital (Table S1). This study was approved by the ethics reviewing committee with an approval number of 18MMHIS187e. Written informed consent was obtained from patients prior to sampling. Clean reads from RNA-Seq were subjected to bioinformatics analysis to determine transcripts per kilobase of transcript per million mapped reads (TPM) [40].

### Plasmids, expression, and reporter assay

Lentiviral vector HR’-VGF for exogenous VGF expression is a kind gift from Prof. Chou, Y.T [24]. Besides, the cDNA of OSCC cells was reversely transcribed at 65℃ using Maxima H Minus Reverse Transcriptase (ThermoFisher Scientific). Then the VGF coding sequence (2.2 Kb) was amplified from cDNA using KAPA HiFi HotStart PCR kit (Roche, Basel, Switzerland) (Table S2). Following digestion with *Sna*BI and *Sal*I, the cleaved fragment was ligated into the pBabe-puro retroviral vector and designated as pBabe-puroVGF. OSCC cell subclones stably express VGF were established after viral infection or plasmid transfection, and puromycin selection. The 1.9 Kb coding sequence of IL23R was amplified from the cDNA of OSCC cell reversely transcribed by MMLV-HP reverse transcriptase (Lucigen, Middleton, WI, USA). Following the digestion of *Bam*HI and *Hind*III, the cleaved fragment was ligated into pcDNA3.1(-) plasmid and designated pcDNA3.1(-)IL23R.

The generation of reporter constructs to denote the targeting of miR-432-5p on the 3’UTR of VGF transcript followed our previous strategy [5]. Table S2 enlisted the primers used to generate reporter constructs.

### Establishment of the CRISPR-dCas9 SAM system

The PAM sequences used for dCas9 recognition, which localize at a position within 200 bp upstream of the transcription start site (TSS) in the VGF gene, were retrieved from the website (sam.genome-engineering.org/). Oligonucleotides containing guide sequences were ligated into sgRNA (MS2) cloning backbone (Cat No: 61,424, Addgene, Watertown, MA, USA) to achieve constructs enabling promoter activation [4]. Upregulation of VGF mRNA expression following transfection designated endogenous induction elicited by SAM system.

### qRT-PCR analysis

SyGreen-based qRT-PCR analysis was performed using the Step OnePlus Real-Time PCR System (Applied Biosystems, Waltham, MA, USA) and VGF or IL23R primers (Table S3). TaqMan miRNA assay system and miR-432-5p probe (Cat. No.: 442,976, Applied Biosystems) was used to assay miR-432-5p expression. GAPDH or RNU6B was used as a control for quantification [4].

### Western blot analysis

Western blot analysis followed our previous protocol [5]. The primary antibodies are listed in Table S4. The proteins in the supernatant were collected from the equivalent amount of cells using the trichloroacetic acid (TCA) protein precipitation method [41]. The signals of VGF in cell lysate were normalized against GAPDH to realize the expression level.

### In silico analysis

Algorithms evaluated, including the Ingenuity Pathway Analysis (IPA, nih.gov/resources/) for functional enrichment and canonical pathway identification, along with algorithms CIBERSOR-ABS, EPIC, MCPCounter, QUANTISEQ, TIMER, and XCELL for TIME annotation. The TCGA (cancer.gov/ccg/research/genome-sequencing/tcga), GEO (Gene Expression Omnibus, ncbi.nlm.nih.gov/geo/), and Encori (rnasysu.com/encori/) portals were utilized to investigate the expression profiles in the database, while TargetScan (targetscan.org/vert_80) and PiTa (https://genie.weizmann.ac.il) were used for studying miRNA-mRNA interaction.

### Statistics

Data were presented as mean ± standard error. Mann-Whitney test, *t-*tests, two-way ANOVA test, and linear correlation analysis were performed. Kaplan-Meier survival analysis was used to assess the overall survival. The prognostic signatures were also analyzed using a multivariate Cox regression module. *ns*, not significant. *, ** and *** represent *p* < 0.05, *p* < 0.01 and *p* < 0.001, respectively.

## Results

### Upregulation of VGF defines the worse tumor prognosis

**Fig. 1:**
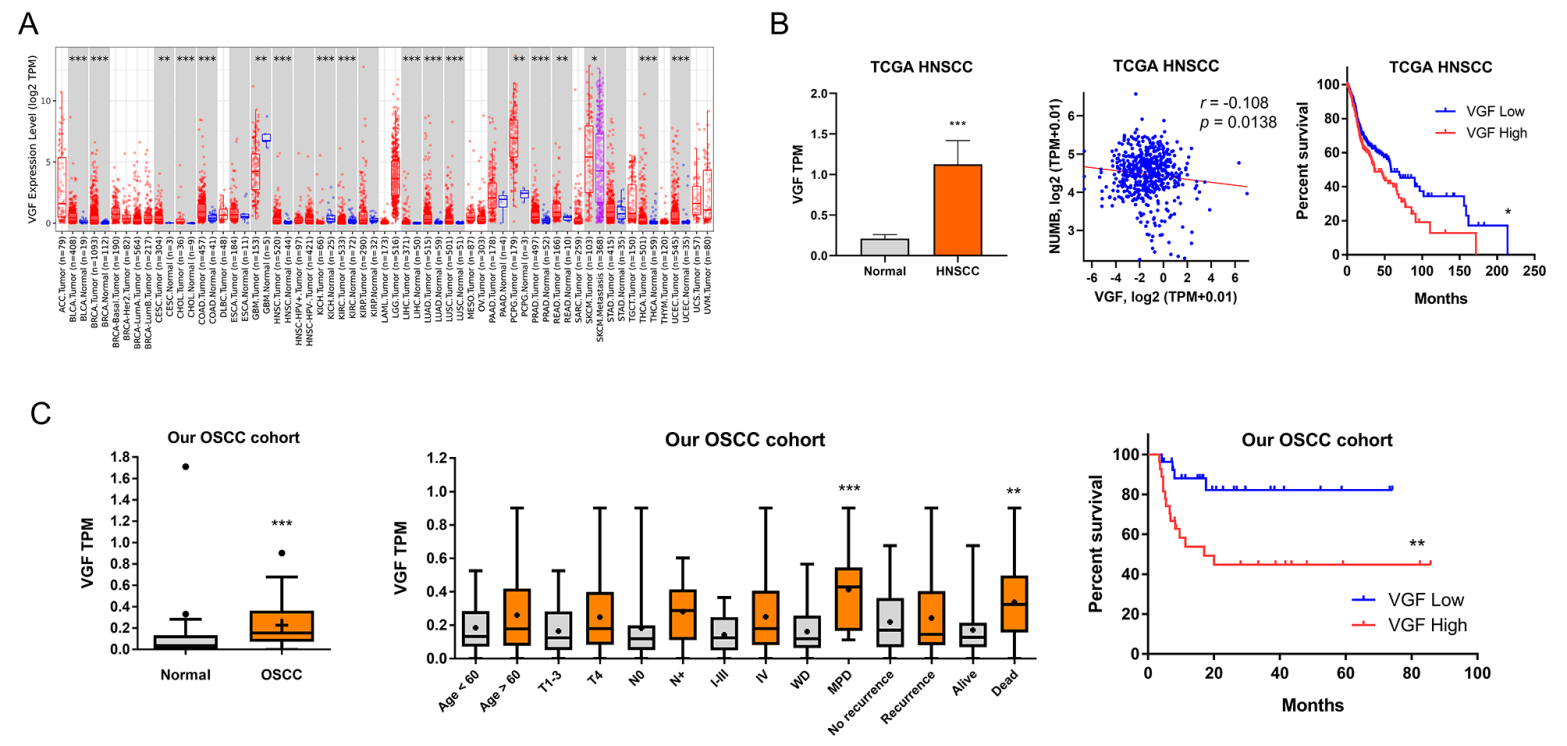
Upregulation of VGF in HNSCC and OSCC. (**A**) Analysis of pan-malignancies in TCGA database using TIMER algorithm. Upregulation of VGF transcript in the vast majority of paired malignancies is noted. (**B**) TCGA HNSCC data. Lt, upregulation of VGF in tumors. Middle, a reverse correlation lies between VGF expression and NUMB expression. Rt, Kaplan-Meier survival curve illustrating an association between high VGF expression and worse patient survival. (**C**) Our OSCC cohort data. Lt, upregulation of VGF in tumors. Middle, VGF expression as related to clinicopathologic parameters. Rt, Kaplan-Meier survival curve illustrating an association between high VGF expression and worse patient survival. Medium TPM values of VGF in HNSCC or OSCC tumors are used as a cutoff to designate high or low expression in VGF

Analysis of TCGA HNSCC tumors using the TIMER program indicated that VGF was significantly upregulated in 20 out of 23 types of malignancies with tissue pairing (Fig. 1A). VGF was also drastically upregulated in HNSCC tumors for approximately four folds compared to normal tissue (Fig. 1B, Lt). VGF expression was opposite to NUMB expression in this tumor cohort, which is compatible with our previous observation (Fig. 1B, Middle). The opposition in VGF and NUMB expression was also noticed in 18/20 tumor types in the TCGA dataset (Table S5). High VGF expression was associated with worse HNSCC patient survival (Fig. 1B, Rt). The analysis of our transcriptome data also revealed the VGF upregulation in OSCC tumors in relation to controls (Fig. 1C, Lt). VGF upregulation was associated with higher tumor grade and higher frequency of patient mortality during follow-up (Fig. 1C, Middle). High VGF expression was associated with worse OSCC patient survival (Fig. 1C, Rt).

### Targeting of miR-432-5p on VGF

**Fig. 2:**
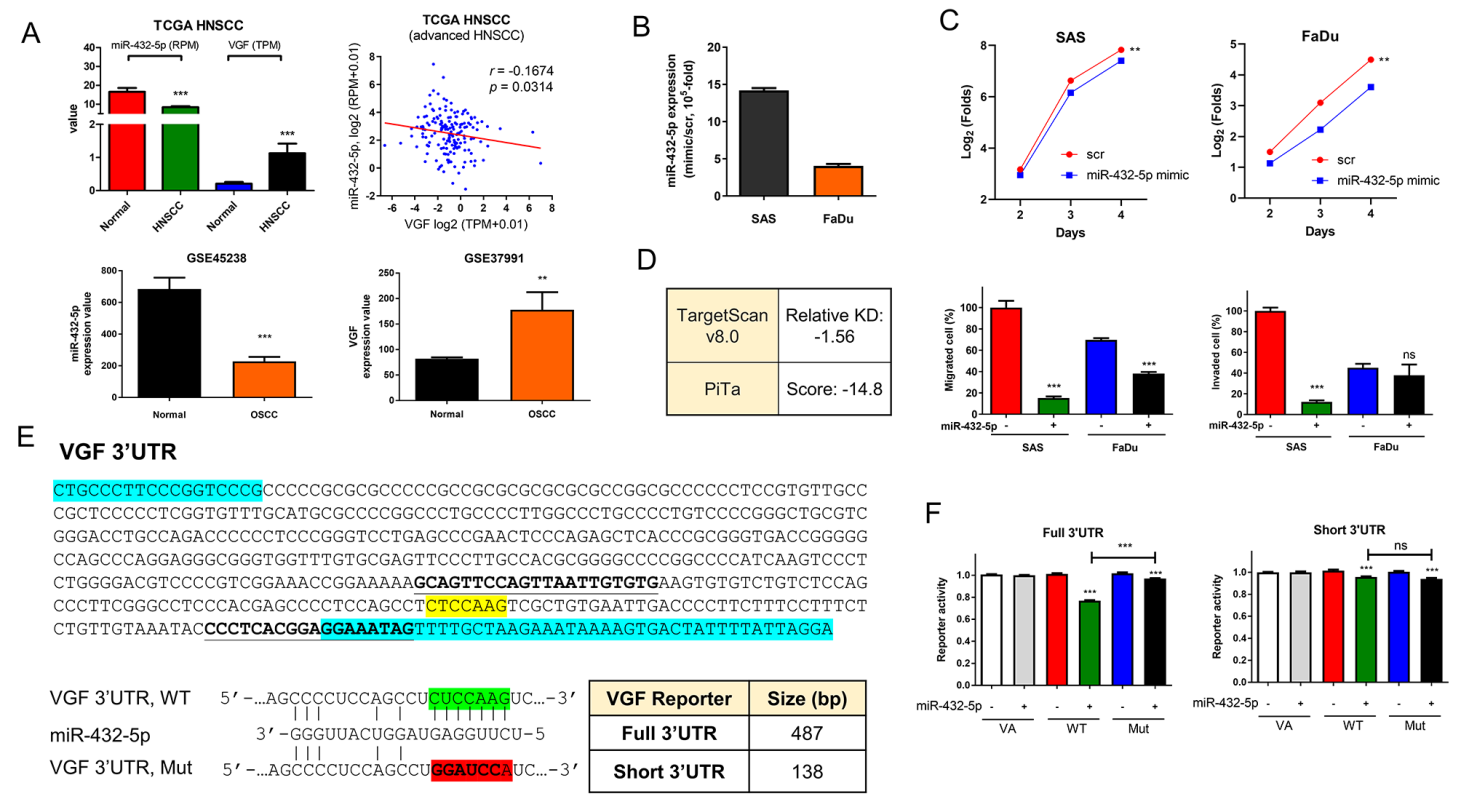
miR-432-5p suppresses oncogenicity and targets VGF. (**A**) HNSCC data. Upper Lt, downregulation of miR-432-5p, and upregulation of VGF expression in tumors. Upper Rt, a reverse correlation lies between VGF expression and miR-432-5p expression in the advanced HNSCC patient subset (T3/T4 and node-positive; *n* = 166). Lower, GEO dataset of Taiwanese OSCC, Lt, downregulation of miR-432-5p in tumors; Rt, upregulation of VGF in tumors. (**B**, **C**) The effects of miR-432-5p mimic in OSCC cells. (**B**) Induction of miR-432-5p expression with the treatment of mimic. (**C**) Phenotypes in OSCC cells. Upper, growth. A two-way ANOVA test is performed to evaluate the difference in growth in the exponential growth phase between day 2 and day 4. The original growth curves are shown in Fig. S1. Lower Lt, migration; Lower Rt, invasion. (**D**) Prediction of targeting of miR-432-5p on the 3’UTR of VGF transcript. (**E**) Upper, VGF 3’UTR sequences. Blue boxes, primer sites to amplify full 3’UTR sequence. Bold fonts/underline, primers to amplify short 3’UTR sequence. Yellow box, predicted targeted site. Lower, Lt, the alignment of miR-432-5p, wild-type and mutant sequences in the reporters. Green box, wild-type targeted sequences. Red box, mutant sequences. Rt, the differences in the size of full 3’UTR reporter and short 3’UTR reporter. (**F**) Reporter assays. Lt, full 3’UTR reporter. Rt, short 3’UTR reporter

Downregulation of miR-432-5p expression was noted in the TCGA HNSCC dataset (Fig. 2A, Lt). miR-432-5p expression was reversely correlated with VGF expression (Fig. 2A, Rt). Downregulation of miR-432-5p and upregulation of VGF expression was also noted in the Taiwanese OSCC database (Fig. 2A, Lower). To investigate the oncogenic potential of miR-432-5p, we transfected SAS and FaDu cells with miR-432-5p mimic to upregulate its expression (Fig. 2B). The induction of miR-432-5p expression decreased growth (Fig. 2C, Upper and Fig. S1), migration (Fig. 2C, Lower Lt) and invasion (Fig. 2C, Lower Rt) in both cells. The inhibitory effects of miR-432-5p on mobility are more prominent in SAS cells, while the growth inhibition mediated by miR-432-5p is more prominent in FaDu cells. TargetScan and PiTa modes predicted the direct targeting of miR-432-5p on the 3’UTR of VGF transcript (Fig. 2D). The 487-bp full 3’UTR reporter and a short 3’UTR reporter, which is a 138-bp segment encompassing target site in 3’UTR, were generated (Fig. 2E, Upper). Mutant reporters generated from these wild-type reporters are also established (Fig. 2E, Lower). The induction of miR-432-5p expression suppressed the activity of wild-type full 3’UTR reporter, while such suppression was largely reversed in mutant reporter (Fig. 2F, Lt). However, the induction of miR-432-5p expression resulted in slight but equivalent suppression of both the wild-type and mutant short 3’UTR reporter (Fig. 2F, Rt). The findings suggest that other sequences localized in 3’UTR apart from the predicted target site might be essential for the negative regulation of miR-432-5p on VGF.

### Transient exogenous expression increases secreted VGF

**Fig. 3:**
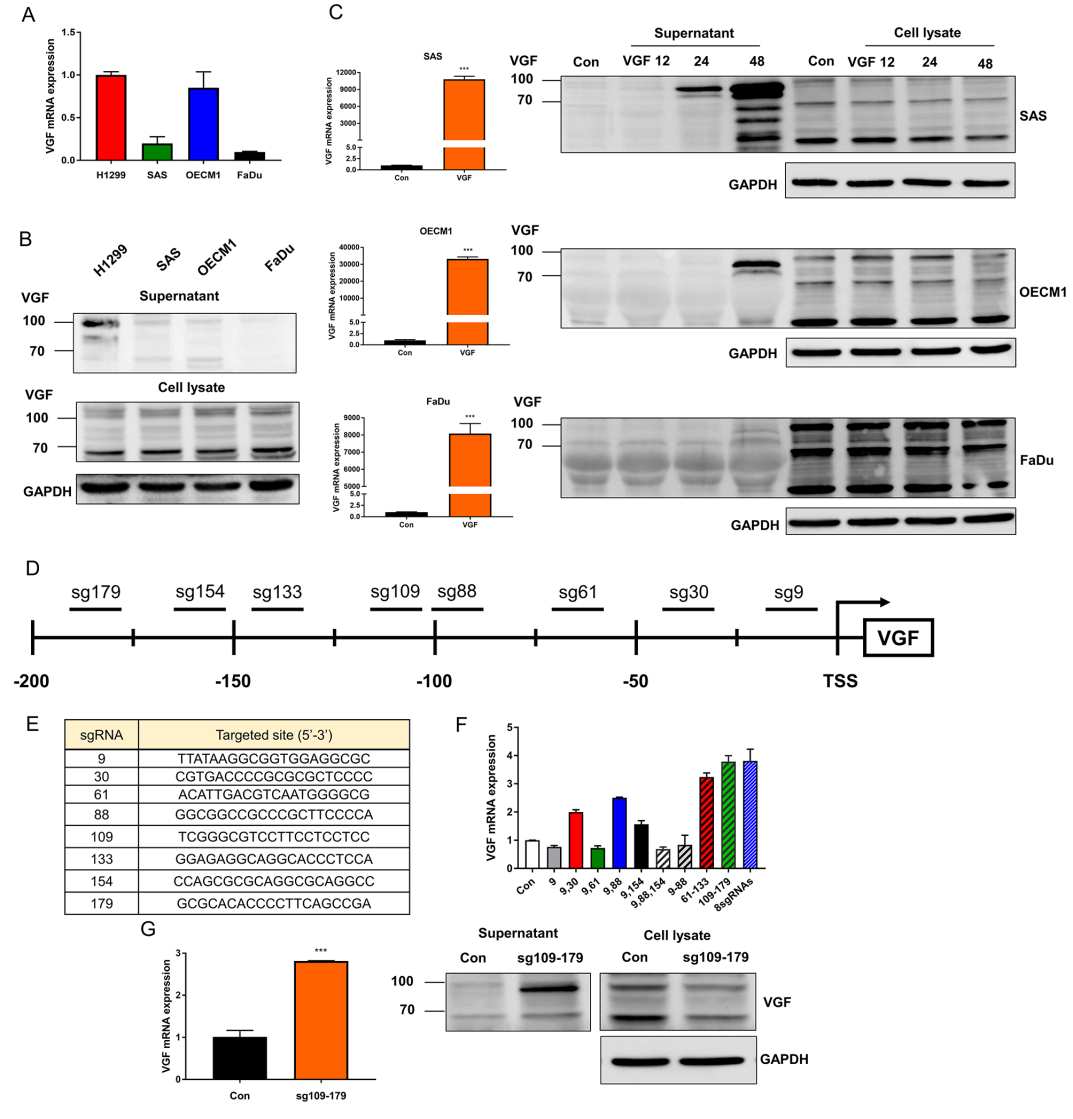
Induction of VGF expression in OSCC cells. (**A**) VGF mRNA expression in cell lines. H1299 serves as a control to denote the relative VGF expression levels in OSCC cells. (**B**) Western blot analysis of supernatant and cell lysate. (**C**) Transient exogenous VGF expression. Upper, SAS; Middle OECM1; Lower, FaDu. Lt, VGF mRNA expression following the transfection for 24 h. Rt, Western blot analysis to show the VGF protein in the supernatant and cell lysate following the transfection for 12, 24, or 48 h. Con, vector alone control; VGF, HR’-VGF plasmid. (**D** – **G**) Induction of endogenous VGF expression using SAM system. (**D**) Schematic diagram to illustrate 9 predicted sgRNA binding segments in VGF promoter that may allow for SAM-based activation. (**E**) sgRNA sequences. (**F**) qPCR analysis. The changes in VGF mRNA expression following SAM induction using solitary sgRNA or combined sgRNAs transfection. The combination of sgRNA 109/133/154/179 yields induction effects simulating the combination of 8 sgRNAs. (**G**) Lt, qPCR analysis. Rt. Western blot analysis. It shows that the induction of VGF mRNA expression is associated with the increased VGF protein in the supernatant 24 h after induction. Con, vector alone control

The H1299 NSCLC cell line exhibiting high VGF expression was used as a control. The VGF mRNA expression is 0.2, 0.8, and 0.1 in SAS, OECM1, and FaDu, relative to H1299, respectively (Fig. 3A). VGF protein in cell lysate, shown as bands at approximately 100, 80, and 65-KDa mobility positions, were identified across different cell lines. Distinctive bands at 95, 80, and 65-KDa positions were evident in the H1299 supernatant. Whereas the abundance of VGF protein was modest in SAS and OECM1 supernatants and scanty in FaDu supernatant (Fig. 3B). The transfection of HR’-VGF plasmid for 24 h rendered the drastic increase of VGF mRNA expression in OSCC cells. However, although the cytosolic VGF did not increase within 48 h, the secreted VGF increased in OSCC cells following infection at 48 h. SAS had the most abundant exogenous VGF in the supernatant, which started at 24 h (Fig. 3C).

### Transient endogenous expression increases secreted VGF

SAM system was imposed to elicit endogenous VGF expression by promoter activation. Eight predicted sites localized in 5′-region of TSS are illustrated in Fig. 3D. Plasmids containing the guide oligonucleotides to bind target sequences and SAM components were generated, and these were named sg9 to sg179 according to the location of the first targeted nucleotide relative to TSS (Fig. 3D, E). The transfection of different plasmid combinations caused the differential VGF activation (Fig. 3F). Following the induction of sg109, sg133, sg154, and sg179, VGF mRNA expression significantly increased (Fig. 3F, G), which was accompanied by a notable increase in secreted VGF (Fig. 3G).

### Increase of growth and migration in cells with stable VGF expression

**Fig. 4:**
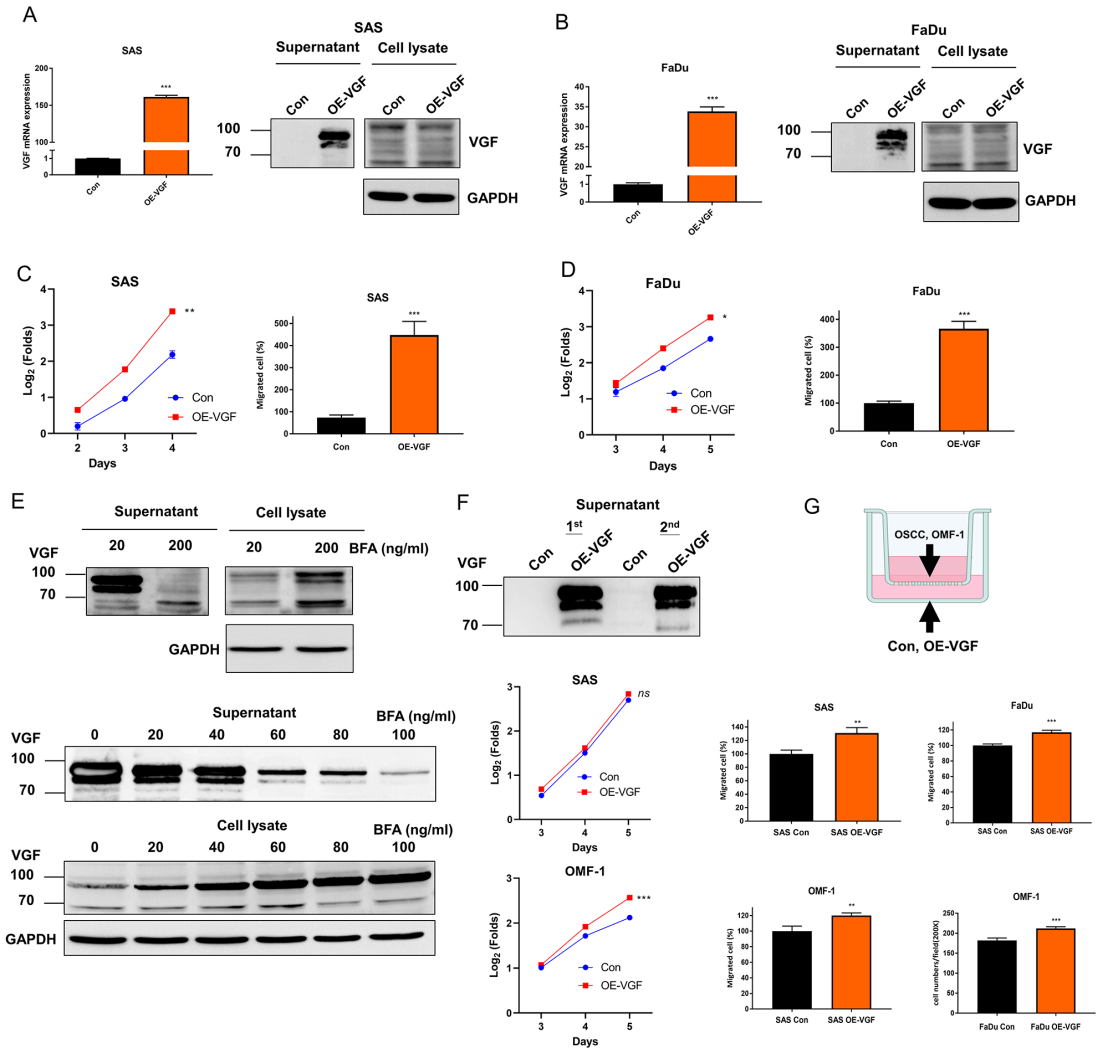
The generation of OSCC OE-VGF stable cells and phenotypic influences. (**A**, **C**) SAS cell. (**B**, **D**) FaDu cell. (**A**, **B**) Lt, qPCR analysis. Rt, Western blot analysis. (**C**, **D**) Lt, growth. A two-way ANOVA test is performed to evaluate the difference in growth in the exponential growth phase between day 2 and day 4 in SAS cells and between day 3 and day 5 in FaDu cells. The original growth curves are shown in Fig. S2. Rt, cell migration assay. Drastic increases in VGF mRNA expression, supernatant VGF protein, growth, and migration are noted in stable cell subclones relative to controls. Con, vector alone control. (**E** – **G**) The effects of supernatant and co-cultivated OSCC OE-VGF. (**E**) The VGF localization in SAS OE-VGF cell following BFA treatment for 24 h. Upper, With the treatment of 200 ng/ml, the secretion of VGF is completely inhibited, and the cytosolic retention of VGF is noted. Lower, the gradual decrease of VGF secretion and accumulation of cytosolic VGF notified following the 0–100 ng/ml BFA treatment. (**F**) Upper, Western blot analysis of the supernatant was collected in two turns during the continuous SAS OE-VGF culture. Middle and Lower, growth. A two-way ANOVA test is performed to evaluate the difference in growth in the exponential growth phase between day 3 and day 5. The original growth curves are shown in Fig. S4. Adding supernatants at day 1 and day 3 to culture barely affects the growth of SAS and significantly accelerates OMF-1 growth. Con, vector alone control. (**G**) Upper, schematic diagram to illustrate the co-culture system. Middle, co-cultivated SAS OE-VGF increases the migration of OSCC cells compared to the control. Lower, co-cultivated OSCC OE-VGF increases the migration of OMF-1 cells compared to the control

Stable OSCC cells were established by selecting cells infected with lentiviruses. Stable VGF expression cells were designated OE-VGF. In SAS and FaDu OE-VGF cell subclones, the increased VGF mRNA expression (Fig. 4A Lt, 4B Lt) was accompanied by increased VGF secretion (Fig. 4A Rt, 4B Rt). SAS OE-VGF exhibited increased growth (Fig. 4C, Lt, and Fig. S2A) and migration (Fig. 4C, Rt) compared to the control. Likewise, the growth (Fig. 4D, Lt, and Fig. S2B) and migration (Fig. 4D, Rt) of FaDu OE-VGF also increased. OECM1 OE-VGF also exhibited an increase in secreted VGF (Fig. S3). The exogenous or endogenously induced VGF proteins were mainly secreted VGF other than cytosolic VGF.

### Secreted VGF from OSCC cells promotes the growth and migration of adjacent OMF

SAS OE-VGF cell subclones were treated with BFA to ascertain the secretion effects to inhibit the secretion. The 200 ng/ml treatment almost abolished the secretion, and the VGF was accumulated in cytosol (Fig. 4E, Upper). The dose effects of BFA in decreasing secreted VGF and increasing cytosolic VGF were also noted (Fig. 4E, Lower). Condition media were collected twice from SAS OE-VGF cells being cultivated in a serum-free medium for 24 h. These aliquots of condition media were added to the cultivation of SAS and OMF-1 on day 1 and day 3, respectively (Fig. 4F, Upper). The conditioned medium from SAS OE-VGF did not affect the growth of SAS cells (Fig. 4F, Middle, and Fig. S4A), due likely to the presence of endogenous VGF in SAS cells. Nevertheless, it significantly accelerated the growth of OMF-1 cells post-day 3 (Fig. 4F Lower and Fig. S4B). The co-culture approach was further imposed to view the paracrine effects of VGF-expressing cells (growing in the lower chamber) on the migration of co-cultivated OSCC cells and OMF-1 cells (growing in the upper chamber; illustrated in Fig. 4G, Upper). SAS OE-VGF increased the migration of co-cultured OSCC cells (Fig. 4G, Middle). OSCC OE-VGF cell subclones also increased the migration of co-cultured OMF-1 cells (Fig. 4G, Lower).

### Identification of VGF-modulated neoplastic functions and effectors

**Fig. 5:**
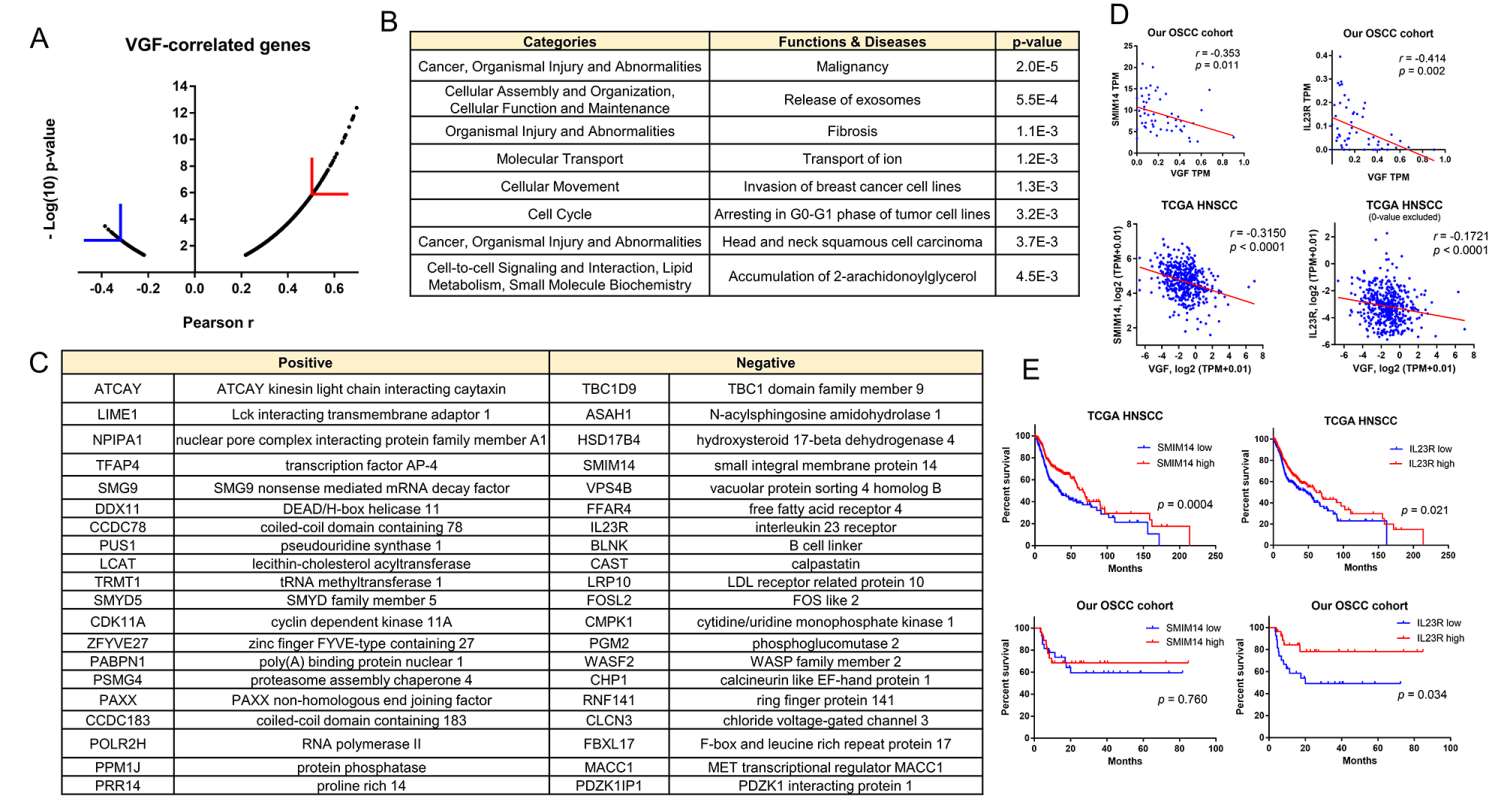
VGF co-regulated genes and functions. (**A**) Dot plot of VGF co-regulated transcripts in our OSCC tumor cohort. X-axis, *r* value; Y-axis, -Log10(*p* value). The number of positively correlated transcripts is much more than those negatively correlated. Symbol ∟ separate *r* > 0.50 or *r* < -0.33 transcripts from the remaining transcripts. (**B**) The functions and diseases associated with VGF co-regulated transcripts analyzed with the IPA algorithm. (**C**) Enlisting of coding transcript highly co-regulated with VGF, as revealed in (**A**). The detailed parameters are organized in Table S7. (**D**) The correlation between VGF expression and smim14 expression (Lt panels), and IL23R expression (Rt panels) in our OSCC tumors (Upper panels, analyzed with TPM) and TCGA HNSCC tumors (Lower panels, analyzed with semi-log graph of TPM+0.01). (**E**) Kaplan-Meier survival analysis. Upper panels, TCGA HNSCC tumors. Lower panels, our OSCC tumors. Medium TPM values of SMIM14 or IL23R in tumor samples are used as cutoffs to designate high or low expression of these genes

From the transcriptome of our OSCC tumor cohort, we retrieved 2121 transcripts significantly co-regulated with the VGF transcript, with *r* > 0.22 or < -0.22, for further analysis (Fig. 5A). The IPA analysis showed that these correlated genes were mainly enriched in malignancies, proliferation, exosome release, ion transport, metabolism, and others. These transcripts were also enriched for breast cancer invasion and HNSCC pathogenesis (Fig. 5B). The algorithm further specified the influence of VGF on metabolism, neural regulation, cell-cell interaction and other canonical pathways (Table S6). Using *r* > 0.50 and *r* < -0.33 as the cutoff, the coding transcripts most eminently co-upregulated or co-downregulated with VGF were further identified (Fig. 5C). Their correlation with VGF was also shown in the TCGA HNSCC dataset (Table S7). Smim14 is a small ER-resident transmembrane protein with an uncertain role in the neoplastic process [42]. Among 40 candidates highly co-regulated with VGF in our OSCC and TCGA HNSCC cohorts (Fig. 5C and D), only smim14 and IL23R were prognostic factors in TCGA HNSCC tumors (Fig. 5E, Upper). A reverse correlation between VGF and smim14 expression was observed across all types of malignancies (18/18) in the TCGA database (Table S8). Likewise, reverse correlation in VGF expression and IL23R expression was also noted in nearly all malignant tumor types (11/12) in the TCGA database (Table S9), including those carcinomas in breast and prostate, NSCLC, and CRC. Our OSCC tumor cohort analysis showed that IL23R, but not smim14, was a prognostic factor (Fig. 5E, Lower). The potential modulation of VGF on TIME addressed by six algorithms signified that high VGF expression in tumors was associated with the general repression in immune cell population scores together with those of endothelium, CAF, and stroma. Alterations in the TIME profile for HNSCC and OSCC, in line with VGF expression, appear very similar (Fig. S5 and Table S10). Owing to the limitation in cohort size, the power of statistical analysis of TIME scores in our OSCC cohort was not evident.

### Exogenous IL23R expression abrogates the mobility phenotypes of VGF

**Fig. 6:**
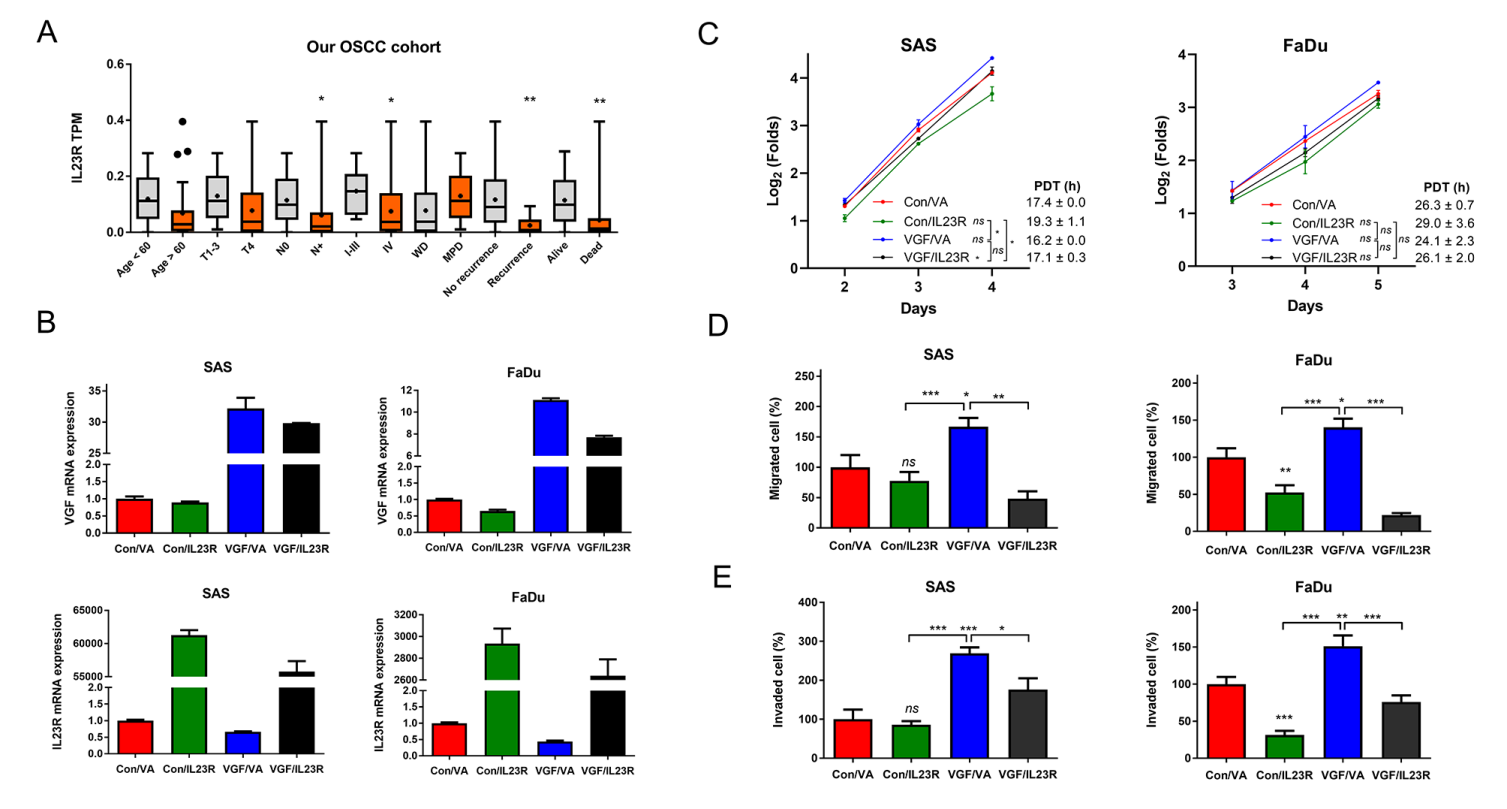
Clinical and functional implications of IL23R expression. (**A**) IL23R transcripts as related to clinicopathological parameters in our OSCC tumors. X-axis clinicopathological states. Y-axis, TPM of IL23R. (**B** – **E**) Exogenous IL23R expression rescues VGF-associated oncogenicity in OSCC cells. (B) qPCR analysis. Duplicate analysis, statistical analysis not performed. (**C** - **E**) Phenotypes. (**C**) Growth. A two-way ANOVA test is performed to evaluate the difference in growth in the exponential growth phase between day 2 and day 4 in SAS cells and between day 3 and day 5 in FaDu cells. The original growth curves are shown in Fig. S6. (**D**) Migration, (**E**) Invasion. Lt, SAS cell. Rt, FaDu cell. VGF, VGF expression cell subclones established by the transfection of pBabe-puroVGF plasmid and selection with puromycin. Con, Control cell subclones. IL23R, Transfection of pcDNA3.1(-)IL23R plasmid. VA, vector alone transfection

Lower IL23R expression was associated with nodal metastasis, advanced stages, and recurrence of OSCC (Fig. 6A). Furthermore, Cox regression analysis signified that VGF, IL23R, or both VGF and IL23R were survival predictors of OSCC (Table 1), subsequent approaches focused on IL23R. A construct containing an IL23R coding sequence was generated to transfect OSCC cell subclones transiently. qPCR analysis detected the changes in VGF or IL23R mRNA expression (Fig. 6B). Of note, the endogenous IL23R expression in VGF-expressing OSCC cell subclones was slightly lower than the control subclones (Fig. 6B). The profile of growth curves and the PDT suggest the faster or slower growth of cells associating with exogenous VGF or IL23R expression, respectively (Fig. 6C and Fig. S6). The migration (Fig. 6D), and invasion (Fig. 6E) were more pronounced in VGF-expressing OSCC cell subclones compared to controls, whereas exogenous IL23R expression mitigated these phenotypes. Moreover, the VGF-enriched mobility phenotypes were rescued by the suppressor activity mediated by IL23R expression. The findings substantiate the existence of a VGF-IL23R regulatory cascade. Of note, in TCGA HNSCC and our OSCC samples, the high IL23R expression was associated with the general increase of immune cell population, endothelium, CAF, and stroma (Table S11), which contrasted to the impacts of VGF.

**Table 1:** Cox regression analysis for survival prediction

	OSCC cohort	
Variable	Hazard ratio	95% CI
N	6.56	2.14 to 20.04
T	1.92	0.56 to 6.65
VGF	4.25	1.40 to 12.92
IL23R	0.34	0.12 to 0.97
VGF and IL23R*	13.57	1.73 to 106.2
	**TCGA HNSCC**	
**Variable**	**Hazard ratio**	**95% CI**
N	1.23	0.93 to 1.60
T	1.06	0.81 to 1.39
VGF	1.23	0.94 to 1.61
IL23R	0.76	0.59 to 0.99
VGF and IL23R*	1.55	1.08 to 2.22

*(VGF^low^ and IL23R^high^) vs. (VGF^high^ and IL23R^low^)

## Discussion

This study identifies the upregulation of VGF expression in OSCC for the first time. VGF upregulation is an unfavorable prognostic factor for OSCC and HNSCC. Although VGF seems to be highly expressed in OSCC with less squamous differentiation, as the upregulation of VGF mRNA expression is also detected in major types of malignancies, VGF abnormality should no longer be considered specific for neural or neuroendocrine malignancies. VGF expression is inducible as responding to extrinsic stimuli or factors such as NGF and BDNF [19]. Studies have shown that binding sites for E-box, CCAAT, CRE, and NFκB in promoter are critical for VGF induction [43, 44]. Besides, SOX9 induces VGF production by transactivating of its binding site at ➔-800 bp location upstream of TSS in the promoter [18]. As SOX9 is involved in the pathogenesis and progression of OSCC and the crosstalks between tumor cells and stromal cells [45, 46], this factor could be one of the stimuli underlying the VGF upregulation in OSCC. The promoter hypermethylation is associated with the silencing of VGF in some epithelial tumors [26]. By way of contrast, with the treatment of HDAC1 inhibitor, VGF expression is induced in lung adenocarcinoma cells [25]. Our SAM strategy induces endogenous VGF mRNA expression through the delivery of transactivating apparatus and sgRNA targeting an approximately 100 bp segment near the TSS of the promoter. The results suggest the potential existence of regulatory elements spanning the − 100 – -200 nucleotide segment in the VGF promoter, which may be associated with VGF upregulation.

miR-432-5p appears to be a crucial core in the circular RNA (circRNA) regulatory loop as many circRNAs, including ciRS7, activate targeted genes by absorbing and repressing miR-432-5p to elicit the oncogenesis [47]. The suppressor activity of miR-432-5p against OSCC has been confirmed in this study [47]. We propose previously unspecified clues demonstrating that VGF is a new target of miR-432-5p. In OSCC, an epigenetic miR-432-5p-VGF interplay exists, in addition to promoter regulation also exists in OSCC [26]. miR-432-5p is a suppressor miRNA being dysregulated in multiple neoplasms [48, 49]. It is also dysregulated in different psycho-neural disorders, including neural malignancies [50]. The values of circRNAs-miR-432-5p-VGF axis in the therapy of neoplasia and psycho-neural pathology deserve comprehensive investigation.

In neural or neuroendocrine cells, VGF polypeptides and various cleaved peptides are processed, packed, and then undergo a programmed secretion pathway [19]. Like neural cells [20], the secretion of VGF from other types of cells also follows similar programs [21, 22]. This study shows that the intrinsic VGF protein or induced VGF protein tends to secret readily into the medium in OSCC cell lines. Only when treated with a secretion inhibitor does the VGF stay more in the cytosol. Although a modest ➔ 70-KDa band, most likely the precursor peptide, and suspicious cleaved fragments are seen in cell lysate or supernatant aliquot, additional bands localized between ~ 80 KDa and 95 KDa mobility positions are also evident, concerns about the artifacts are raised. However, since the same electrophoretic signals in H1299 cells are detected in our side-by-side experiment, and the VGF state in H1299 has been relatively well defined [24], these high molecular weight bands are considered authentic.

Given that the size of these bands is beyond that of the precursor polypeptide, it is postulated that the structural modification or the complexing with other molecules would have occurred to facilitate their exportation. We have validated the function of secreted components in mediating paracrine modulation on OSCC cells or stromal cells. Despite the active constituents within the released material and the exportation machinery awaiting analysis, our findings substantiate that VGF is a growth or oncogenic factor in the broad spectrum of tissues beyond the restriction in the neuroendocrine system.

In addition to the modulation of function and tumor progression in OSCC, the potential consequence of VGF on immune suppression being disclosed was consistent with previous studies [30–32]. The roles of the candidate effectors that were retrieved, especially smim14, in oncogenesis and immune regulation require stratification. IL23R is involved in the immune-editing of laryngeal cancer cells for drug resistance and survival [37]. However, treatment with IL23 and TGFβ inhibitor attenuates the progression of precancers to become OSCC in murine models [36]. Although the complicated molecular mechanisms are unresolved, this study provides clues in tissue analysis, clinical data stratification, and functional assay substantiating that IL23R is an antitumor downstream effector of VGF. As a cytokine receptor, our findings also highlight the association between IL23R expression and immune enrichment in counteracting OSCC progression. Although regulating VGF on the IL23-IL23R signal axis in modulating TIME for OSCC progression requires further elucidation, the interception of the VGF-IL23R cascade in OSCC tumors may bestow therapeutic efficacy.

## Conclusion

This work specifies that the paracrine activities of VGF modulate oncogenic potential, tumor microenvironment, and immune responses during OSCC pathogenesis. The interplays of miR-432-5p-VGF-IL23R regulatory axis and associated molecular networks would be crucial diagnostic markers or therapeutic targets in neoplasms as VGF upregulation is present in nearly all types of malignancies in addition to OSCC or HNSCC.

### Electronic supplementary material

Below is the link to the electronic supplementary material.

Supplementary Material 1

Supplementary Material 2

## Data Availability

The datasets used and/or analyzed during the current study are available from the corresponding author upon reasonable request.

## Abbreviations

CRC: Colorectal carcinoma
HNSCC: Head and neck squamous cell carcinoma
IL23: Interleukin 23
NSCLC: Non-small cell lung carcinoma
OSCC: Oral squamous cell carcinoma
TSS: Transcription start site
VGF: Nerve growth factor inducible
