# Supplementary figures and images for: The upregulation of VGF enhances the progression of oral squamous carcinoma
Source: Cancer Cell Int. 2024 Mar 25;24:115. doi: 10.1186/s12935-024-03301-9 (PMC10964619; doi:10.1186/s12935-024-03301-9)

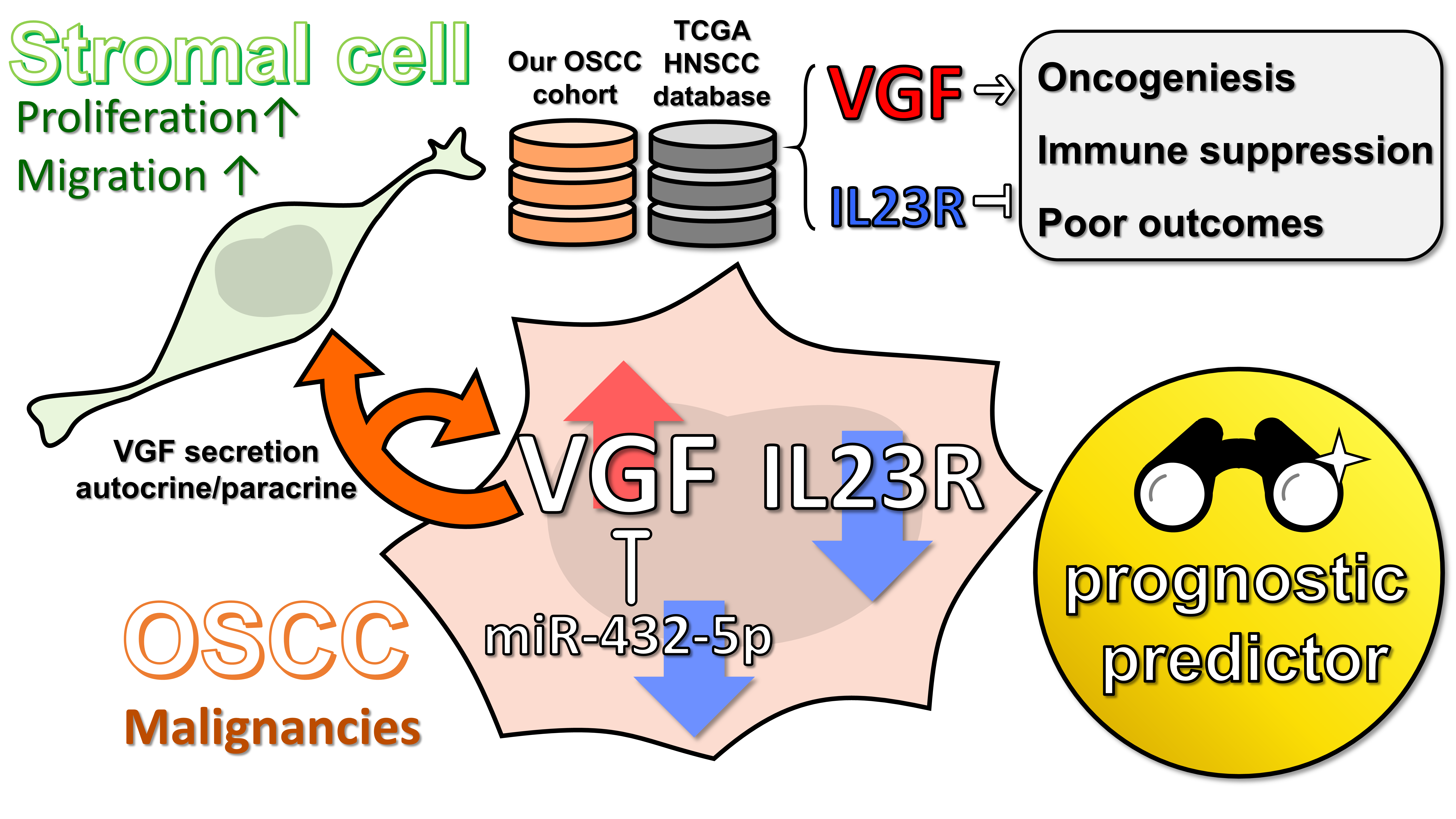

Supplement: Supplementary file 1 — Supplementary Material 1 [file 12935_2024_3301_MOESM1_ESM.png]
