# Supplementary material for: The upregulation of VGF enhances the progression of oral squamous carcinoma
Source: Cancer Cell Int. 2024 Mar 25;24:115. doi: 10.1186/s12935-024-03301-9 (PMC10964619; doi:10.1186/s12935-024-03301-9)
Supplement: Supplementary file 2 — Supplementary Material 2 [file 12935_2024_3301_MOESM2_ESM.docx]

**Supplementary Figure S1:**

**
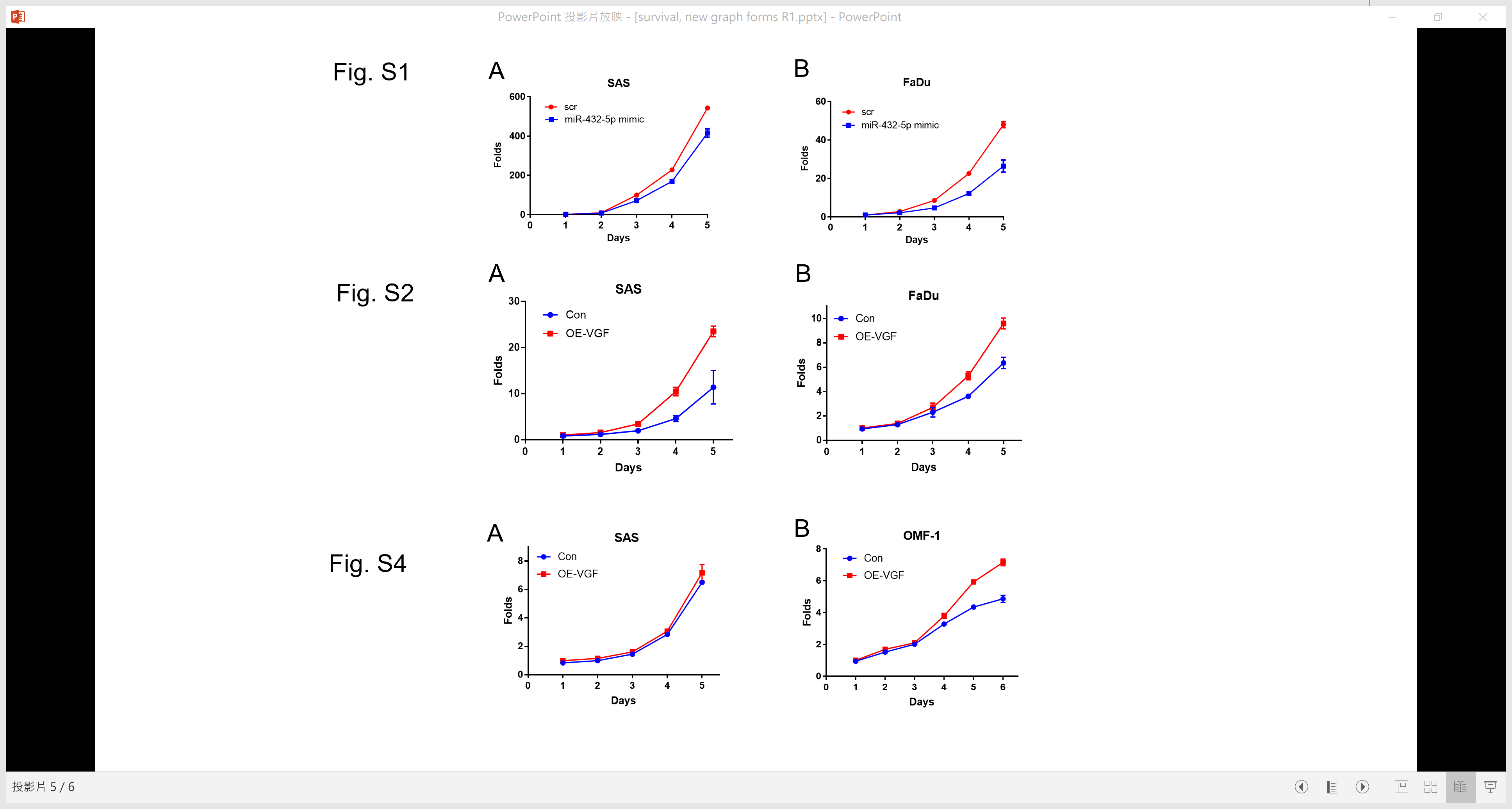
**Growth of OSCC cells with the treatment of miR-432-5p mimic. (A) SAS. (B) FaDu.

**Supplementary Figure S2:**

Growth of OSCC cells with stable VGF overexpression (OE-VGF). (A) SAS. (B) FaDu.


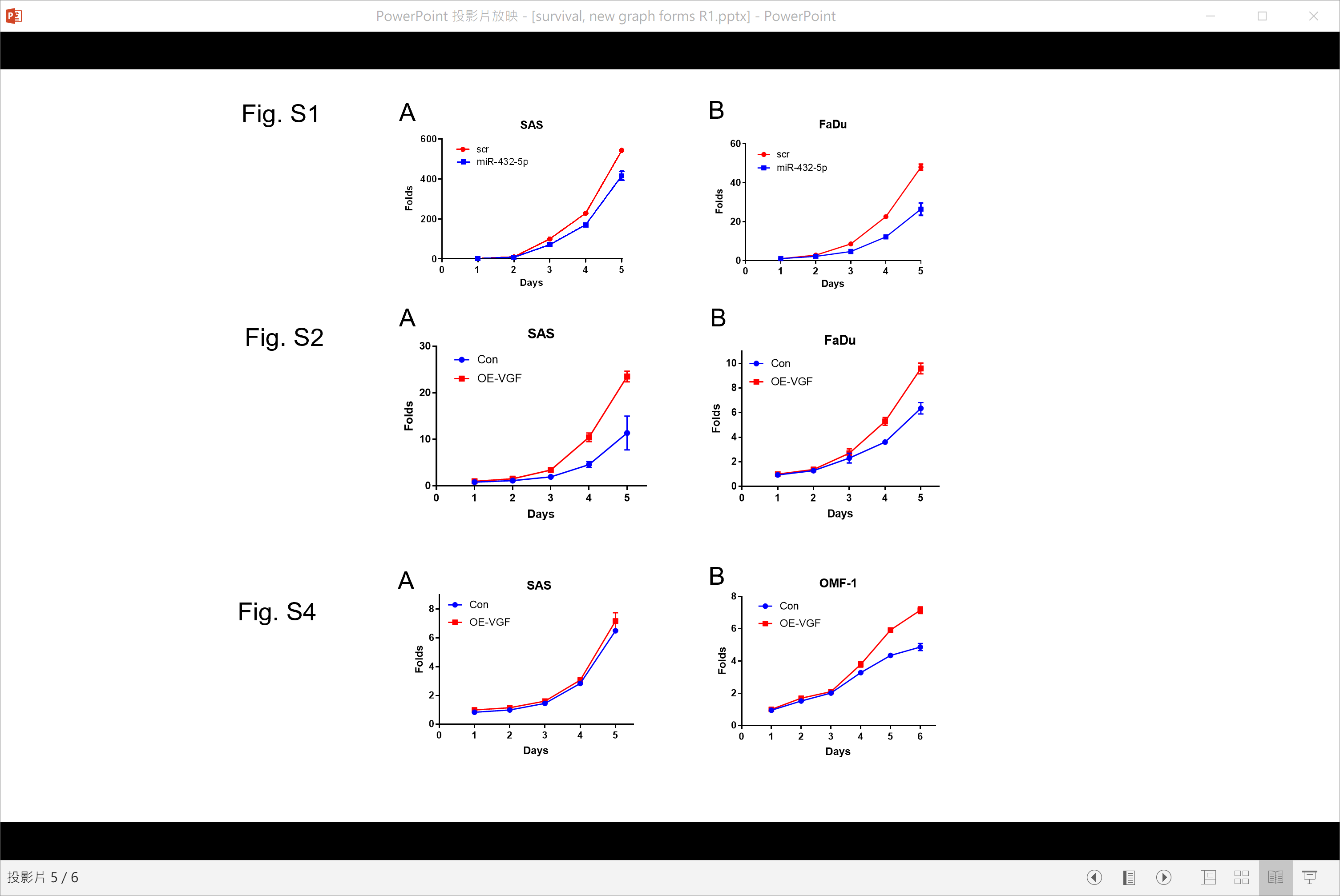


**Supplementary Figure S3:**

The generation of OECM1 OE-VGF stable cell subclone. (A) qPCR analysis. (B) Western blot analysis of VGF protein in supernatant and cell lysate.


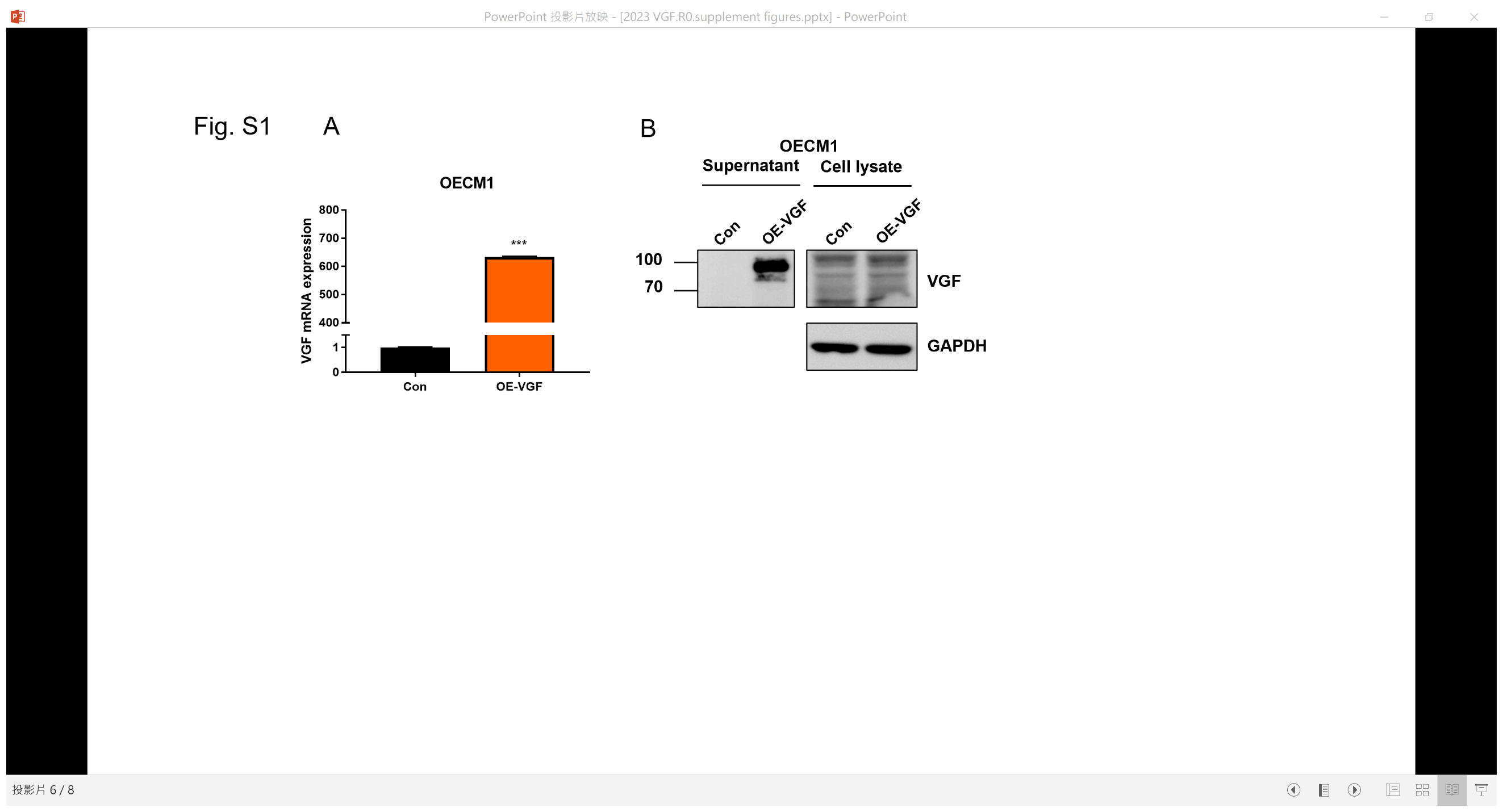


**Supplementary Figure S4:**

Growth of cells in medium with the aliquots of SAS OE-VGF condition medium. (A) SAS. (B) OMF-1.


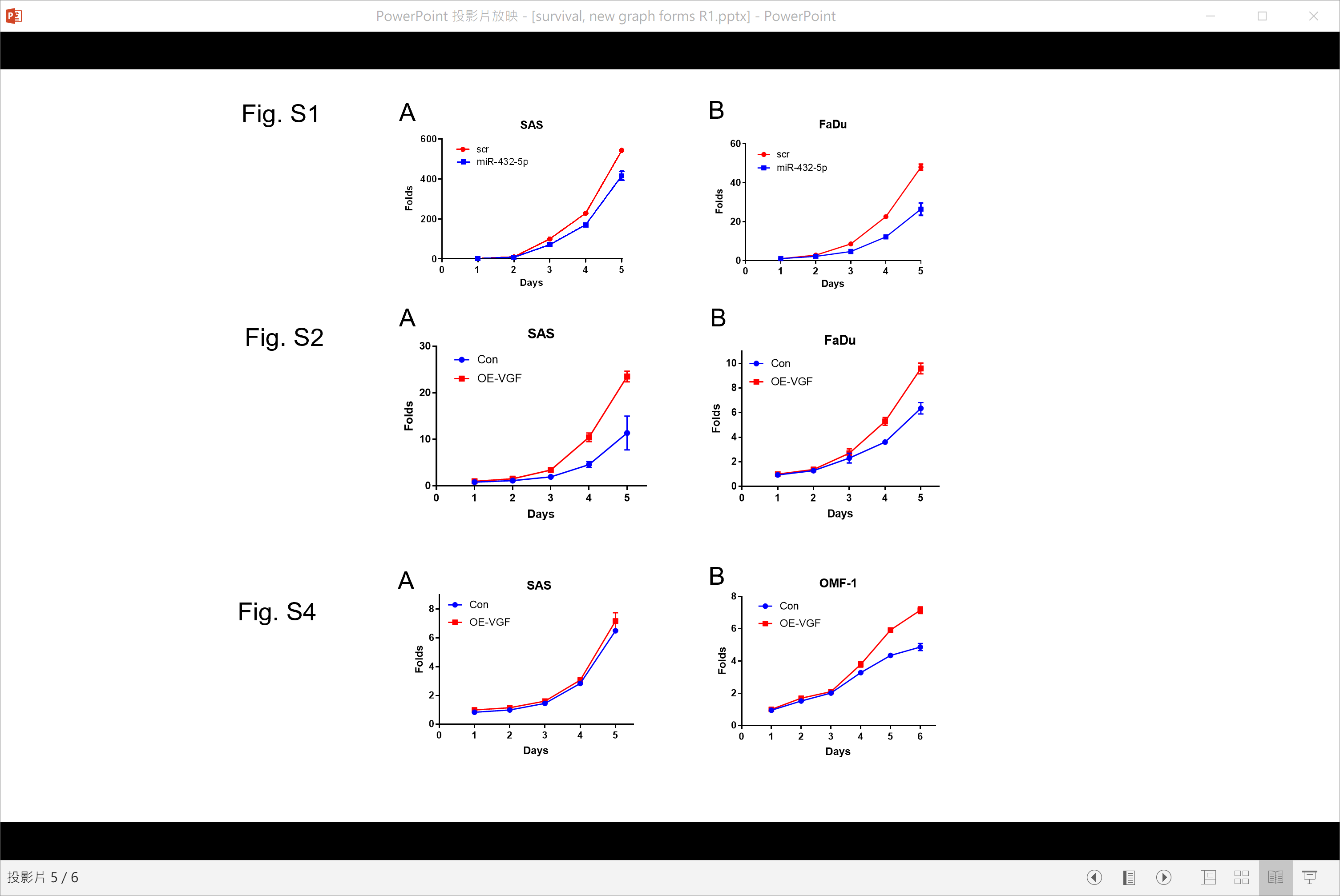


**Supplementary Figure S5:**

The individual results of CIBERSORT-ABS, EPIC, MCPCOUNTER, QUANTISEQ, TIMER, and XCELL analysis. They indicate the association between VGF expression and TIME in TCGA HNSCC tumors. X-axis, cell types or TIME states; Y-axis, score. Blue bars, decrease; red bars, increase. L, low VGF expression. H, high VGF expression according to the medium value of VGF FPKM-UQ


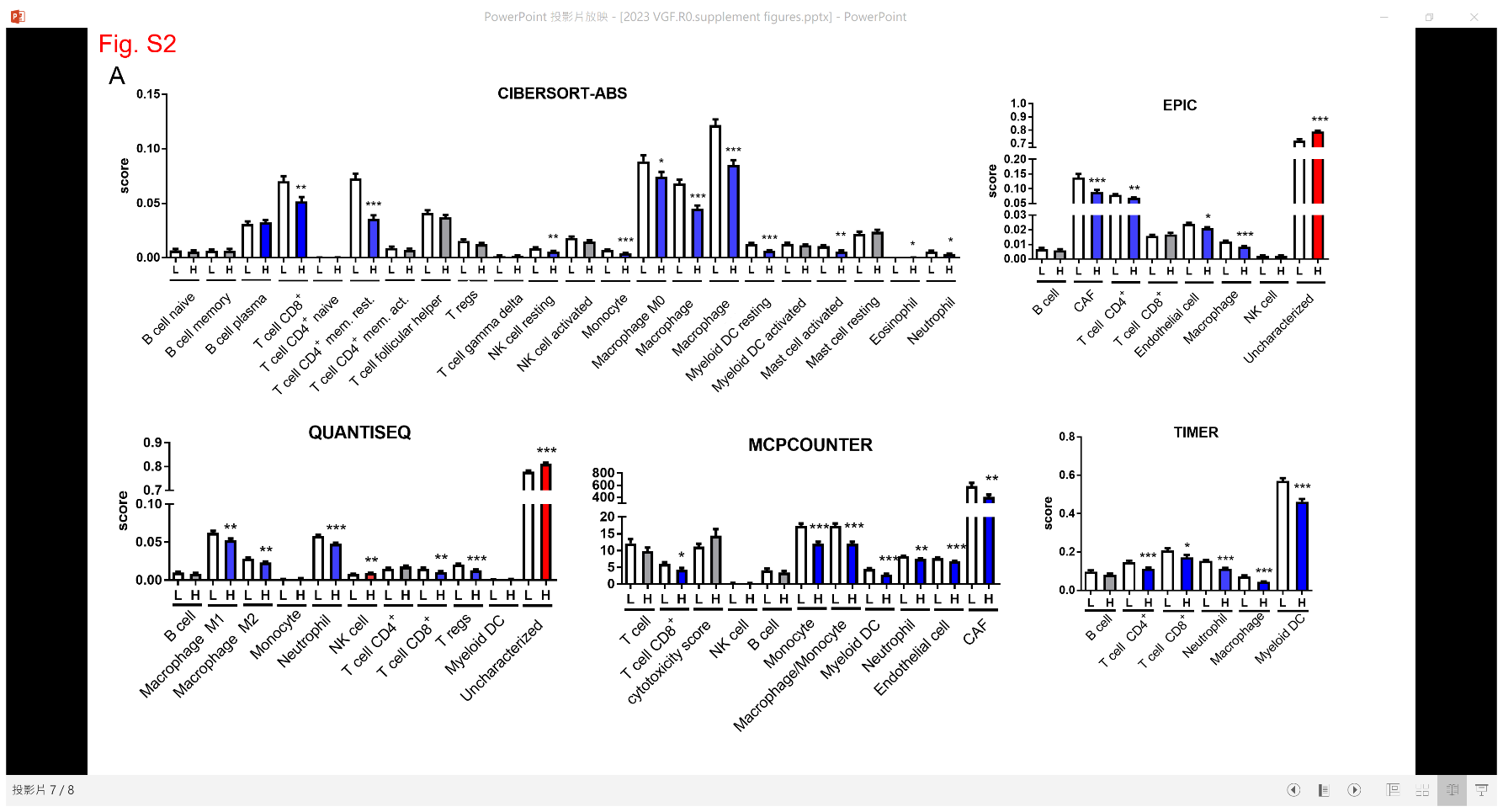


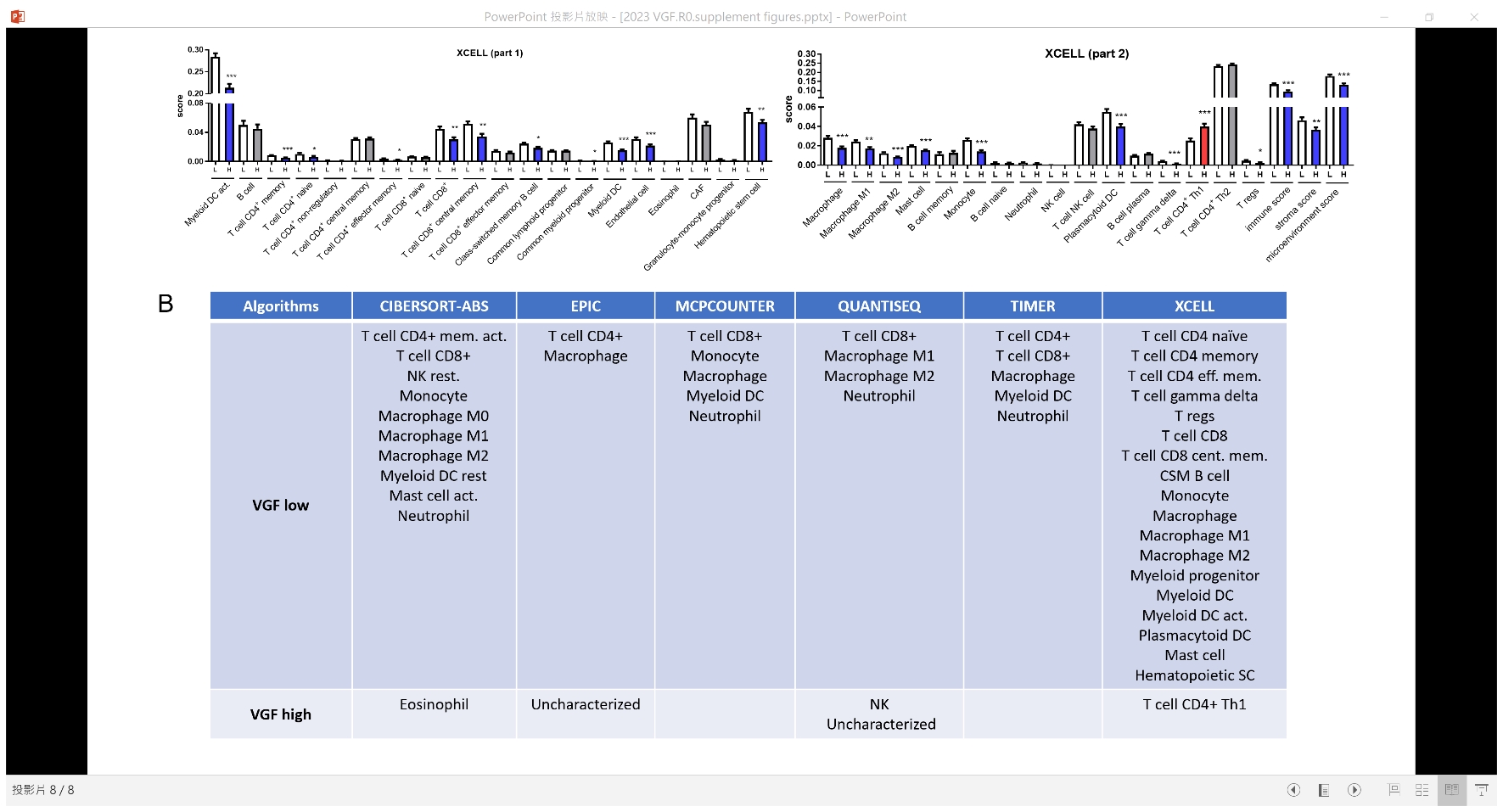


**Supplementary Figure S6:**

The growth of OSCC cells as related to the VGF and IL23R expression. (A) SAS. (B) FaDu.


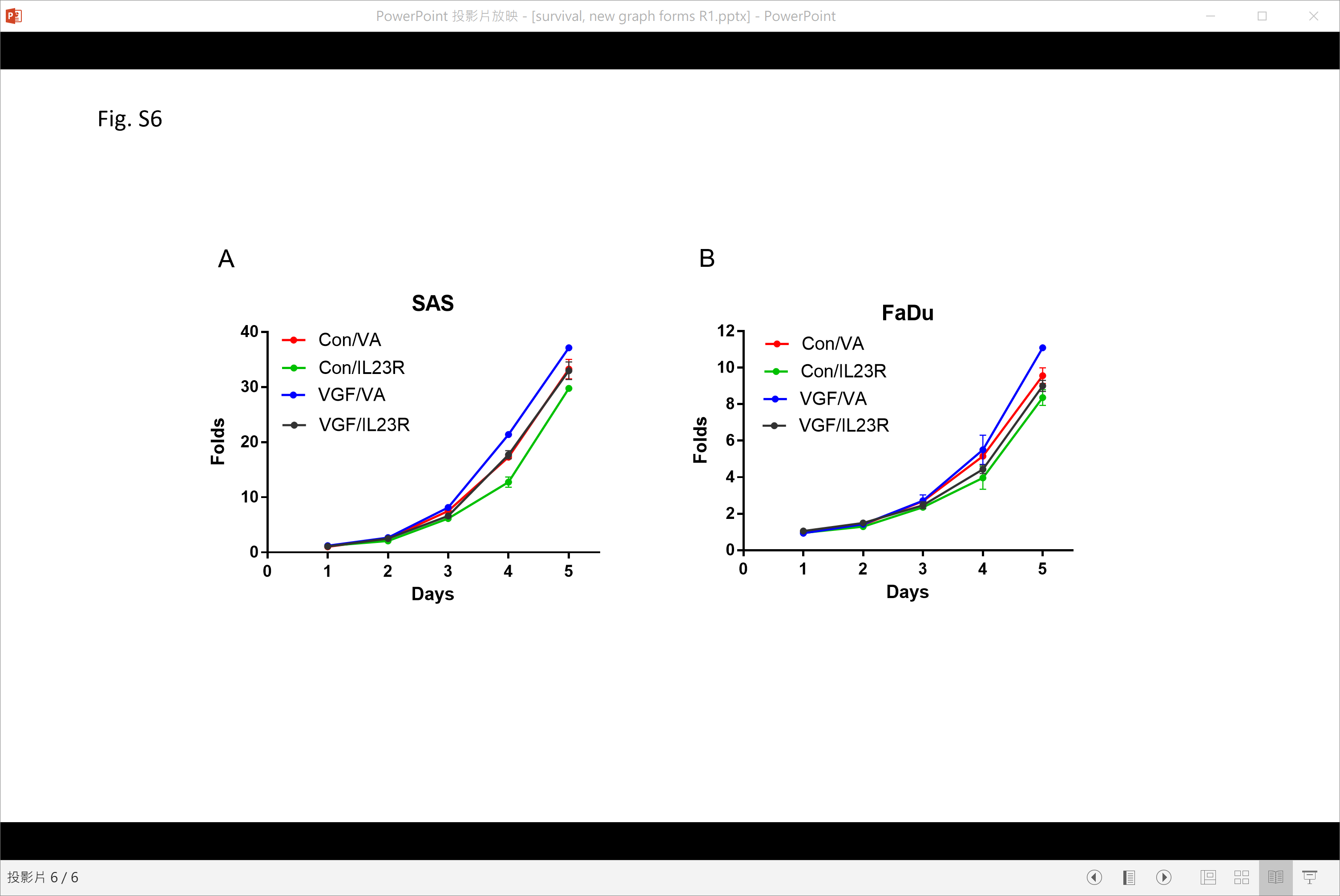


**Supplementary Table S1:**

Clinicopathological parameters of our OSCC cohort.

| **Characteristics** | | **No. of cases** | **%** |
| --- | --- | --- | --- |
| Gender | Male | 52 | 96.3 |
|  | Female | 2 | 3.7 |
| Age | ≧ 60 | 30 | 55.6 |
|  | < 60 | 24 | 44.4 |
| Oral habits | Yes | 49 | 90.7 |
|  | No | 5 | 9.3 |
| T | 1-3 | 14 | 25.9 |
|  | 4 | 40 | 74.1 |
| N | 0 | 30 | 55.6 |
|  | 1, 2 | 24 | 44.4 |
| Stage | I-III | 12 | 22.2 |
|  | IV | 42 | 77.8 |
| Differentiation | Well | 40 | 74.1 |
|  | Moderate-Poor | 14 | 25.9 |
| Recurrence | Yes | 15 | 27.8 |
|  | No | 39 | 72.2 |
| Survival | Yes | 36 | 66.7 |
|  | No | 18 | 33.3 |

**Supplementary Table S2:**

Primers used for PCR-based cloning

| Primer | 5’ - 3’ |
| --- | --- |
| VGF-F | CCCTACGTAGACCCTCCTCTCCACCTCTC |
| VGF-R | CCCGTCGACAAGGGAACTCGCACAAACCA |
| IL23R-F | CGAGGATCCTTCCAGACATGAATCAGGTC |
| IL23R-R | GGGAAGCTTACCACACAGCTCTACTTTTCC |
| VGF-reporter full-F | CTTACTAGTCTGCCCTTCCCGGTCCCG |
| VGF-reporter full-R | CCGAAGCTTCCTAATAAAATAGTCACTTTTATTTCTTAGCAAAACTATTTCC |
| VGF-reporter short-F | GCCACTAGTGCAGTTCCAGTTAATTGTGTG |
| VGF-reporter short-R | GGGAAGCTTCTATTTCCTCCGTGAGGG |

F, forward primer; R, reverse primer

**Supplementary Table S3:**

Primers for qPCR analysis

| Primer | 5’ - 3’ |
| --- | --- |
| VGF-F | CCTTCCCGAAACCCACAAGTT |
| VGF-R | GCCTTGGTACGCCTTGGAC |
| IL23R-F | AGCAGCAATTAAGAACTGCCA |
| IL23R-R | TACCAAAGCCGAGCTGTTGTT |
| GAPDH-F | TGGTATCGTGGAAGGACTCATGAC |
| GAPDH-R | ATGCCAGTGAGCTTCCCGTTCAGC |

F, forward primer; R, reverse primer

**Supplementary Table S4:**

Antibodies used in this study

| Name | Company | Cat No. |
| --- | --- | --- |
| Anti-VGF antibody | Abcam | Ab74140 |
| Anti-GAPDH (6C5) | Santa Cruz Biotech | sc-32233 |

**Supplementary Table S5:**

Correlation between NUMB and VGF expression (negative in 18/20)

| **Cancer** | | ***r*** | ***p*** |
| --- | --- | --- | --- |
| TGCT | Testicular Germ Cell Tumors | -0.52 | 4.5E-12 |
| BRCA | Breast Invasive Carcinoma | -0.41 | 3.9E-46 |
| DLBC | Lymphoid Neoplasm Diffuse Large B-cell Lymphoma | -0.41 | 4.1E-03 |
| BLCA | Bladder Urothelial Carcinoma | -0.36 | 2.6E-14 |
| PRAD | Prostate Adenocarcinoma | -0.33 | 2.8E-14 |
| UCEC | Uterine Corpus Endometrial Carcinoma | -0.31 | 2.9E-13 |
| THYM | Thymoma | -0.28 | 1.9E-03 |
| HNSCC | Head and Neck Squamous Cell Carcinoma | -0.27 | 1.1E-09 |
| PAAD | Pancreatic Adenocarcinoma | -0.24 | 1.3E-03 |
| SKCM | Skin Cutaneous Melanoma | -0.20 | 1.4E-05 |
| KIRC | Kidney Renal Clear Cell Carcinoma | -0.19 | 8.8E-06 |
| CESC | Cervical Squamous Cell Carcinoma and Endocervical Adenocarcinoma | -0.18 | 2.0E-03 |
| STAD | Stomach Adenocarcinoma | -0.17 | 7.2E-04 |
| LUAD | Lung Adenocarcinoma | -0.17 | 7.4E-05 |
| COAD | Colon Adenocarcinoma | -0.17 | 2.4E-04 |
| LGG | Brain Lower Grade Glioma | -0.17 | 9.4E-05 |
| LIHC | Liver Hepatocellular Carcinoma | -0.13 | 9.2E-03 |
| THCA | Thyroid Carcinoma | -0.12 | 6.7E-03 |
| PCPG | Pheochromocytoma and Paraganglioma | 0.15 | 4.8E-02 |
| CHOL | Cholangiocarcinoma | 0.42 | 1.1E-02 |

**Supplementary Table S6:**

VGF-associated canonical pathways

| **Rank** | **Ingenuity Canonical Pathways** |
| --- | --- |
| 1 | Nicotine Degradation II |
| 2 | Catecholamine Biosynthesis |
| 3 | nNOS Signaling in Neurons |
| 4 | Nicotine Degradation III |
| 5 | Glycogen Biosynthesis II (from UDP-D-Glucose) |
| 6 | Phosphatidylethanolamine Biosynthesis II |
| 7 | Granulocyte Adhesion and Diapedesis |
| 8 | NAD Phosphorylation and Dephosphorylation |
| 9 | Tight Junction Signaling |
| 10 | Guanosine Nucleotides Degradation III |

**Supplementary Table S7:**

Leading VGF co-regulated genes

| **VGF-correlated**  **genes** | **Description** | **Our OSCC**  **cohort** | | **TCGA**  **HNSCC cohort** | |
| --- | --- | --- | --- | --- | --- |
|  |  | ***r*** | ***p*** | ***r*** | ***p*** |
| ATCAY | ATCAY kinesin light chain interacting caytaxin | 0.64 | 1.4E-10 | 0.15 | 9.6E-04 |
| LIME1 | Lck interacting transmembrane adaptor 1 | 0.61 | 1.2E-09 | 0.35 | 6.6E-15 |
| NPIPA1 | nuclear pore complex interacting protein family member A1 | 0.58 | 9.4E-09 | 0.33 | 8.3E-14 |
| TFAP4 | transcription factor AP-4 | 0.56 | 3.9E-08 | 0.25 | 9.4E-09 |
| SMG9 | SMG9 nonsense mediated mRNA decay factor | 0.55 | 8.3E-08 | 0.29 | 8.0E-11 |
| DDX11 | DEAD/H-box helicase 11 | 0.55 | 1.0E-07 | 0.31 | 1.3E-12 |
| CCDC78 | coiled-coil domain containing 78 | 0.54 | 2.1E-07 | 0.48 | 1.0E-30 |
| PUS1 | pseudouridine synthase 1 | 0.53 | 2.8E-07 | 0.49 | 8.5E-32 |
| LCAT | lecithin-cholesterol acyltransferase | 0.53 | 3.6E-07 | 0.31 | 6.4E-13 |
| TRMT1 | tRNA methyltransferase 1 | 0.52 | 5.2E-07 | 0.49 | 7.7E-32 |
| SMYD5 | SMYD family member 5 | 0.52 | 6.0E-07 | 0.23 | 2.9E-07 |
| CDK11A | cyclin dependent kinase 11A | 0.52 | 6.7E-07 | 0.15 | 7.5E-04 |
| ZFYVE27 | zinc finger FYVE-type containing 27 | 0.51 | 9.3E-07 | 0.30 | 1.5E-11 |
| PABPN1 | poly(A) binding protein nuclear 1 | 0.51 | 1.1E-06 | 0.45 | 9.5E-27 |
| PSMG4 | proteasome assembly chaperone 4 | 0.51 | 1.2E-06 | 0.38 | 4.1E-19 |
| PAXX | PAXX non-homologous end joining factor | 0.51 | 1.3E-06 | 0.48 | 1.8E-30 |
| CCDC183 | coiled-coil domain containing 183 | 0.50 | 1.7E-06 | 0.41 | 3.8E-22 |
| POLR2H | RNA polymerase II | 0.50 | 1.8E-06 | 0.44 | 6.7E-25 |
| PPM1J | protein phosphatase | 0.50 | 1.9E-06 | 0.35 | 1.7E-15 |
| PRR14 | proline rich 14 | 0.50 | 2.0E-06 | 0.35 | 2.8E-16 |
| TBC1D9 | TBC1 domain family member 9 | -0.39 | 3.3E-04 | -0.20 | 8.5E-06 |
| ASAH1 | N-acylsphingosine amidohydrolase 1 | -0.37 | 5.8E-04 | -0.14 | 1.7E-03 |
| HSD17B4 | hydroxysteroid 17-beta dehydrogenase 4 | -0.36 | 9.7E-04 | -0.28 | 1.2E-03 |
| SMIM14 | small integral membrane protein 14 | -0.35 | 1.1E-02 | -0.32 | 1.2E-13 |
| VPS4B | vacuolar protein sorting 4 homolog B | -0.35 | 1.2E-03 | -0.31 | 1.4E-12 |
| **VGF-correlated**  **genes** | **Description** | **Our OSCC**  **cohort** | | **TCGA**  **HNSCC cohort** | |
|  |  | ***r*** | ***p*** | ***r*** | ***p*** |
| FFAR4 | free fatty acid receptor 4 | -0.35 | 1.3E-03 | -0.16 | 2.2E-04 |
| IL23R | interleukin 23 receptor | -0.34 | 1.9E-03 | -0.16 | 3.0E-04 |
| BLNK | B cell linker | -0.34 | 2.0E-03 | -0.23 | 3.6E-07 |
| CAST | calpastatin | -0.34 | 2.0E-03 | -0.31 | 1.2E-12 |
| LRP10 | LDL receptor related protein 10 | -0.33 | 2.1E-03 | -0.34 | 9.2E-15 |
| FOSL2 | FOS like 2 | -0.33 | 2.2E-03 | -0.30 | 1.3E-11 |
| CMPK1 | cytidine/uridine monophosphate kinase 1 | -0.33 | 2.2E-03 | -0.22 | 8.8E-07 |
| PGM2 | phosphoglucomutase 2 | -0.33 | 2.3E-03 | -0.20 | 1.8E-12 |
| WASF2 | WASP family member 2 | -0.33 | 2.4E-03 | -0.28 | 3.0E-10 |
| CHP1 | calcineurin like EF-hand protein 1 | -0.33 | 2.5E-03 | -0.30 | 4.0E-12 |
| RNF141 | ring finger protein 141 | -0.33 | 2.6E-03 | -0.21 | 1.3E-06 |
| CLCN3 | chloride voltage-gated channel 3 | -0.33 | 2.7E-03 | -0.30 | 3.9E-12 |
| FBXL17 | F-box and leucine rich repeat protein 17 | -0.33 | 2.8E-03 | -0.28 | 3.7E-10 |
| MACC1 | MET transcriptional regulator MACC1 | -0.33 | 2.8E-03 | -0.14 | 1.2E-03 |
| PDZK1IP1 | PDZK1 interacting protein 1 | -0.33 | 2.9E-03 | -0.18 | 5.7E-05 |

**Supplementary Table S8:**

Correlation between VGF and SMIM14 expression (positive: negative = 0: 18)

| **Cancer** | | ***r*** | ***p*** |
| --- | --- | --- | --- |
| BLCA | Bladder Urethral Carcinoma | -0.097 | 4.90E-02 |
| BRCA | Breast Invasive Carcinoma | -0.343 | 6.62E-32 |
| COAD | Colon Adenocarcinoma | -0.266 | 4.84E-09 |
| HNSCC | Head and Neck Squamous Cell Carcinoma | -0.323 | 1.16E-13 |
| KIRC | Kidney Renal Clear Cell Carcinoma | -0.265 | 4.58E-10 |
| KIRP | Kidney Renal Papillary Cell Carcinoma | -0.350 | 9.52E-10 |
| LGG | Brain Lower Grade Glioma | -0.249 | 6.04E-09 |
| LIHC | Liver Hepatocellular Carcinoma | -0.311 | 9.03E-10 |
| LUAD | Lung Adenocarcinoma | -0.229 | 1.04E-07 |
| LUSC | Lung Squamous Cell Carcinoma | -0.137 | 2.12E-03 |
| OV | Ovarian Serous Cystadenocarcinoma | -0.212 | 3.12E-05 |
| PRAD | Prostate Adenocarcinoma | -0.349 | 1.06E-15 |
| READ | Rectum Adenocarcinoma | -0.244 | 1.48E-03 |
| SARC | Sarcoma | -0.271 | 8.58E-06 |
| SKCM | Skin Cutaneous Melanoma | -0.293 | 9.00E-11 |
| STAD | Stomach Adenocarcinoma | -0.181 | 4.67E-04 |
| THYM | Thymoma | -0.242 | 8.13E-03 |
| UCEC | Uterine Corpus Endometrial Carcinoma | -0.318 | 2.49E-14 |

**Supplementary Table S9:**

Correlation between VGF and IL23R expression (positive: negative = 1: 11)

| **Cancer** | | ***r*** | ***p*** |
| --- | --- | --- | --- |
| BLCA | Bladder Urothelial Carcinoma | -0.126 | 1.03E-02 |
| BRCA | Breast Invasive Carcinoma | -0.116 | 1.06E-04 |
| COAD | Colon Adenocarcinoma | -0.273 | 1.73E-09 |
| HNSCC | Head and Neck Squamous Cell Carcinoma | -0.155 | 5.00E-04 |
| KICH | Kidney Chromophobe | 0.439 | 2.56E-04 |
| KIRP | Kidney Renal Papillary Cell Carcinoma | -0.167 | 4.33E-03 |
| LUAD | Lung Adenocarcinoma | -0.182 | 2.70E-05 |
| LUSC | Lung Squamous Cell Carcinoma | -0.141 | 1.50E-03 |
| PRAD | Prostate Adenocarcinoma | -0.190 | 1.84E-05 |
| READ | Rectum Adenocarcinoma | -0.161 | 3.73E-02 |
| TGCT | Testicular Germ Cell Tumors | -0.381 | 9.08E-07 |
| THYM | Thymoma | -0.188 | 4.11E-02 |

**Supplementary Table S10:**

VGF expression and TIME scores in TCGA HNSCC and our OSCC

| **Algorithm** | **Cell type** | **HNSCC** | **OSCC** | **Algorithm** | **Cell type** | **HNSCC** | **OSCC** |
| --- | --- | --- | --- | --- | --- | --- | --- |
| CIBERSORT-ABS | T cell CD8+ | * |  | XCELL | Myeloid dendritic cell activated | *** |  |
|  | T cell CD4+ memory resting | *** |  |  | T cell CD4+ memory resting | ** |  |
|  | T regs | * |  |  | T cell CD4+ naïve | * |  |
|  | Macrophage M0 | * |  |  | T cell CD4+ central memory | * |  |
|  | Macrophage M1 | ** |  |  | T cell CD4+ effector memory | *** |  |
|  | Macrophage M2 | *** |  |  | T cell CD8+ naïve | ** |  |
|  | Myeloid dendritic cell resting | *** |  |  | T cell CD8+ | *** |  |
|  | Mast cell activated | * |  |  | T cell CD8+ central memory | ** |  |
| EPIC | Cancer associated fibroblast | ** |  |  | Class-switched memory B cell | ** |  |
|  | Macrophage | *** |  |  | Common myeloid progenitor | ** |  |
|  | Uncharacterized cell | *** |  |  | Myeloid dendritic cell | *** |  |
| MCPCOUNTER | T cell | * |  |  | Endothelial cell | ** |  |
|  | T cell CD8+ | * |  |  | Eosinophil | * |  |
|  | Cytotoxicity score | * |  |  | Macrophage | *** |  |
|  | Monocyte | *** |  |  | Macrophage M1 | *** |  |
|  | Macrophage/Monocyte | *** |  |  | Macrophage M2 | *** |  |
|  | Myeloid dendritic cell | *** |  |  | Mast cell | *** |  |
|  | Cancer associated fibroblast | ** |  |  | Monocyte | *** |  |
| QUANTISEQ | Macrophage M2 | * |  |  | Neutrophil | ** |  |
|  | Neutrophil | *** |  |  | T cell NK | *** |  |
|  | NK cell | ** |  |  | Plasmacytoid dendritic cell | *** |  |
|  | T cell CD4+ (non-regulatory) | ** |  |  | T cell gamma delta | ** |  |
|  | T cell CD8+ | * |  |  | T cell CD4+ Th1 | ** | ** |
|  | T regs | *** |  |  | T cell CD4+ Th2 | ** |  |
|  | Uncharacterized cell | ** |  |  | T regs | *** |  |
| TIMER | T cell CD4+ | ** |  |  | Immune score | *** |  |
|  | Neutrophil | *** |  |  | Stroma score | * |  |
|  | Macrophage | ** |  |  | Microenvironment score | *** |  |
|  | Myeloid dendritic cell | *** |  |  | Granulocyte-monocyte progenitor |  | * |

Red, increase; blue, decrease; empty box, *ns*

**Supplementary Table S11:**

IL23R expression and TIME scores in TCGA HNSCC and our OSCC

| **Algorithm** | **Cell type** | **HNSCC** | **OSCC** | **Algorithm** | **Cell type** | **HNSCC** | **OSCC** |
| --- | --- | --- | --- | --- | --- | --- | --- |
| CIBERSORT-ABS | T cell CD8+ | *** | * | TIMER | B cell | *** | ** |
|  | T reg | *** | * |  | Macrophage | *** | * |
|  | Macrophage M2 | *** | * |  | Myeloid dendritic cell resting | *** | ** |
|  | B cell naïve | *** |  |  | T cell CD4+ | *** |  |
|  | B cell memory | * |  |  | T cell CD8+ | *** |  |
|  | B cell plasma | *** |  |  | Neutrophil | *** |  |
|  | T cell CD4 memory resting | *** |  | XCELL | B cell | *** | ** |
|  | T cell CD4 memory activated | *** |  |  | T cell CD4+ memory | *** | ** |
|  | T cell follicular helper | *** |  |  | T cell CD4+ (non-regulatory) | *** | * |
|  | NK cell resting | ** |  |  | Class-switched memory B cell | *** | * |
|  | NK cell activated | * |  |  | Macrophage M1 | ** | * |
|  | Monocyte | *** |  |  | Mast cell | *** | * |
|  | Macrophage M0 | *** |  |  | B cell memory | ** | ** |
|  | Macrophage M1 | *** |  |  | Monocyte | *** | * |
|  | Myeloid dendritic cell activated | * |  |  | T cell NK | *** | ** |
|  | Mast cell activated | ** |  |  | Plasmacytoid dendritic cell | *** | * |
|  | Neutrophil | ** |  |  | T cell gamma delta | *** | * |
| EPIC | B cell | *** | * |  | T regs | *** | * |
|  | T cell CD4+ | *** | ** |  | Immune score | *** | ** |
|  | Endothelial cell | *** |  |  | microenvironment score | *** | * |
|  | Macrophage | *** |  |  | Myeloid dendritic cell activated | *** |  |
|  | uncharacterized cell | *** |  |  | T cell CD4+ naïve | *** |  |
| MCPCOUNTER | T cell CD8+ | *** | * |  | T cell CD4+ central memory | ** |  |
|  | B cell | *** | ** |  | T cell CD8+ | *** |  |
|  | Monocyte | *** | ** |  | T cell CD8+ central memory | *** |  |
|  | Macrophage/Monocyte | *** | ** |  | T cell CD8+ effector memory | * |  |
|  | T cell | * |  |  | Common myeloid progenitor | ** |  |
|  | NK cell | *** |  |  | Myeloid dendritic cell | *** |  |
|  | Myeloid dendritic cell | *** |  |  | Endothelial cell | *** |  |
|  | Neutrophil | ** |  |  | Granulocyte-monocyte progenitor | *** |  |
|  | Endothelial cell | *** |  |  | Hematopoietic stem cell | *** |  |
| QUANTISEQ | Macrophage M2 | *** | * |  | Macrophage | ** |  |
|  | T cell CD8+ | *** | * |  | B cell naïve | ** |  |
|  | T regs | *** | ** |  | B cell plasma | ** |  |
|  | uncharacterized cell | *** | ** |  | T cell CD4+ Th1 | *** |  |
|  | B cell | *** |  |  | Stroma score | ** |  |
|  | Macrophage M1 | *** |  |  |  |  |  |

Red, increase; blue, decrease; empty box, *ns*.
